# O-Glycosylation Landscapes of SARS-CoV-2 Spike Proteins

**DOI:** 10.3389/fchem.2021.689521

**Published:** 2021-09-06

**Authors:** Yong Zhang, Wanjun Zhao, Yonghong Mao, Yaohui Chen, Shanshan Zheng, Wei Cao, Jingqiang Zhu, Liqiang Hu, Meng Gong, Jingqiu Cheng, Hao Yang

**Affiliations:** ^1^ Key Laboratory of Transplant Engineering and Immunology, MOH, Frontiers Science Center for Disease-related Molecular Network, Institutes for Systems Genetics, West China Hospital, Sichuan University, Chengdu, China; ^2^ Department of Thyroid Surgery, West China Hospital, Sichuan University, Chengdu, China; ^3^ Institute of Thoracic Oncology, West China Hospital, Sichuan University, Chengdu, China

**Keywords:** SARS-CoV-2, spike protein, O-glycosylation, mass spectrometry, EThcD fragmentation

## Abstract

The densely glycosylated spike (S) proteins that are highly exposed on the surface of severe acute respiratory syndrome coronavirus 2 (SARS-CoV-2) facilitate viral attachment, entry, and membrane fusion. We have previously reported all the 22 *N*-glycosites and site-specific *N*-glycans in the S protein protomer. Herein, we report the *O-*glycosylation landscapes of SARS-CoV-2 S proteins, which were characterized through high-resolution mass spectrometry. Following digestion with trypsin and trypsin/Glu-C, and de-*N-*glycosylation using PNGase F, we determined the GalNAc-type *O-*glycosylation pattern of S proteins, including *O*-glycosites and the six most common *O*-glycans occupying them, *via* Byonic identification and manual validation. Finally, 255 intact *O*-glycopeptides composed of 50 peptides sequences and 43 *O*-glycosites were discovered by higher energy collision-induced dissociation (HCD), and three *O*-glycosites were confidently identified by electron transfer/higher energy collision-induced dissociation (EThcD) in the insect cell-expressed S protein. Most glycosites were modified by non-sialylated *O*-glycans such as HexNAc(1) and HexNAc(1)Hex (1). In contrast, in the human cell-expressed S protein S1 subunit, 407 intact *O*-glycopeptides composed of 34 peptides sequences and 30 *O*-glycosites were discovered by HCD, and 11 *O*-glycosites were unambiguously assigned by EThcD. However, the measurement of O-glycosylation occupancy hasn’t been made. Most glycosites were modified by sialylated *O*-glycans such as HexNAc(1)Hex (1)NeuAc (1) and HexNAc(1)Hex (1)NeuAc (2). Our results reveal that the SARS-CoV-2 S protein is an *O*-glycoprotein; the *O*-glycosites and *O*-glycan compositions vary with the host cell type. These comprehensive *O*-glycosylation landscapes of the S protein are expected to provide novel insights into the viral binding mechanism and present a strategy for the development of vaccines and targeted drugs.

## Introduction

The spike (S) protein of severe acute respiratory syndrome coronavirus 2 (SARS-CoV-2) is an extensively *N*-glycosylated protein (Watanabe et al., 2020) that protrudes from the virus surface and binds to the angiotensin-converting enzyme 2 (ACE2) receptor on host cells to mediate cell entry (Wrapp et al., 2020). All 22 *N*-glycosites and *N*-glycans attached to asparagine (Asn, N) in a recombinant S protein protomer expressed in human and insect cells have been identified using high-resolution liquid chromatography–tandem mass spectrometry (LC-MS/MS) (Lenza et al., 2020; Rosenbalm et al., 2020; Walls et al., 2020; Xu et al., 2020; Yan et al., 2020; Zhang et al., 2020; Wang et al., 2021; Zhou et al., 2021). These *N*-glycosites are preferentially distributed in two functional subunits responsible for receptor binding (S1 subunit) and membrane fusion (S2 subunit) (Zhang et al., 2020). Site-specific *N-*glycosylation analysis can provide valuable insights into the infection mechanism and present a strategy for the development of vaccines (Grant et al., 2020).

Unlike *N*-glycosylation, *O*-glycosylation is initiated by the *α*-glycosidic attachment of *N*-acetylgalactosamine (GalNAc) to the hydroxyl group of serine (Ser, S) or threonine (Thr, T), which contains eight types of core structures (Core-1 to Core-8 *O*-glycans), and is involved in a variety of biological functions, such as the mediation of pathogenic binding to human receptors (Mayr et al., 2018; Shajahan et al., 2020a). Moreover, *O-*glycosylation can influence proteolysis during antigen processing, which could prevent the formation of glycopeptides for further presentation to major histocompatibility complex (MHC) and the elicitation of immune response (Wolfert and Boons, 2013). The S protein *O*-glycosites of SARS-CoV-2 have been predicted using computational analysis (Uslupehlivan and Sener, 2020), and Shajahan et al. (2020) identified two *O*-glycosites (T323 and S325) using LC-MS/MS (Shajahan et al., 2020b). However, *O-*glycosylation often occurs in a cluster. Hence, we believe that there are many *O*-glycosites that have not been discovered as deciphering protein *O-*glycosylation remains a big challenge. The comprehensive *O-*glycosylation analysis cannot be performed without appropriate sample preprocessing, analysis methods, and software (King et al., 2017; Qin et al., 2017; Yang et al., 2017; Yang et al., 2018; Ye et al., 2019; Park et al., 2020; Dong et al., 2021).

In the present study, we characterized the intact *O-*glycopeptides of recombinant SARS-CoV-2 S proteins expressed in human and insect cells, using LC-MS/MS. Based on a complementary enzyme digestion strategy, we identified large-scale *O*-glycosites and their corresponding *O*-glycans in the recombinant S proteins. The heterogeneity by different glycoforms of S protein S1 subunits expressed in human and insect cells was resolved and compared. Detailed *O-*glycosylation profiles of S proteins are complementary to the *N-*glycosylation profiles and may help in the development of vaccines and therapeutic drugs.

## Experimental Section

### Materials and Chemicals

Dithiothreitol (DTT), iodoacetamide (IAA), formic acid (FA), trifluoroacetic acid (TFA), Tris base, and urea were purchased from Sigma (St. Louis, MO, United States). Acetonitrile (ACN) was purchased from Merck (Darmstadt, Germany). Zwitterionic hydrophilic interaction liquid chromatography (ZIC-HILIC) materials were purchased from Fresh Bioscience (Shanghai, China). The C18 and C8 membrane were purchased from Agela Technologies (Tianjin, China). Recombinant SARS-CoV-2 S protein (S1+S2 ECD, His tag) expressed by insect cells (High Five) *via* a baculovirus, and S protein (S1, His tag) expressed by human embryonic kidney (HEK293) cells were purchased from Sino Biological (Beijing, China). Codon-optimized DNA sequences encoding the SARS-CoV-2 S protein subunits were cloned into pCMV3-C-His and a baculovirus vector with a poly-histidine tag at the C terminus for recombinant expression of these proteins in human and insect cells, respectively. Sequencing-grade trypsin and Glu-C were obtained from Enzyme & Spectrum (Beijing, China). A quantitative colorimetric peptide assay kit was purchased from Thermo Fisher Scientific (Waltham, MA, United States). Deionized water was prepared using a Milli-Q system (Millipore, Bedford, MA, United States). All other chemicals and reagents of the best available grade were purchased from Sigma-Aldrich or Thermo Fisher Scientific.

### Protein digestion

Recombinant S proteins were proteolyzed using an in-solution protease digestion protocol. In brief, 50 μg of protein were dissolved in 100 μl of 50 mM NH_4_HCO_3_ buffer (pH = 8.5) and heated to denature for 10 min at 95°C. After reduction by DTT (20 mM) for 45 min at 56°C and alkylation with IAA (50 mM) for 1 h at 25°C in the dark, 2 μg of protease (trypsin or trypsin/Glu-C (w/w = 1:1)) was added to the tube and incubated for 16 h at 37°C. Peptides were loaded in a pipette tip which was packed with a C18 membrane. After washing three times using 70 μl of 2% acetonitrile/98% water/0.1% formic acid. Peptides bound to the C18 membrane were eluted three times with 70 μl of 80% acetonitrile/20% water/0.1% formic acid. The peptide concentration was determined using a peptide assay kit, based on the absorbance measured at 480 nm. The peptide mixtures were freeze-dried for further analysis.

### Enrichment of Intact Glycopeptides and N-Glycan Removal

Intact *N*- and *O*-glycopeptides were enriched with ZIC-HILIC materials. Specifically, 20 μg of peptides was suspended in 100 μl of 80% ACN/0.2% TFA solution. 5 mg of Zic-HILIC was washed three times for 10 min each with 0.1% TFA and 80% ACN/0.2% TFA, and 2 mg of processed ZIC-HILIC materials was added to the peptide solution and incubated for 2 h at 37°C. Finally, the mixture was transferred to a 200 μl pipette tip packed with a C8 membrane, and washed twice with 80% ACN/0.2% TFA. After enrichment, intact glycopeptides were eluted thrice with 70 μl of 0.1% TFA, and dried using a SpeedVac concentrator. The enriched intact glycopeptides were digested using 1 U PNGase F dissolved in 50 μl of 50 mM NH_4_HCO_3_ for 2 h at 37°C. The reaction was terminated by adding 0.1% FA. The de-*N*-glycopeptides and *O*-glycopeptides were dried using a SpeedVac concentrator for further analysis.

### Liquid chromatography-Tandem Mass Spectrometry Analysis

All the samples were analyzed using higher energy collision-induced dissociation (HCD) in mass spectrometry (Orbitrap Fusion Lumos mass spectrometer). In brief, intact *O*-glycopeptides and de-*N*-glycopeptides were dissolved in 0.1% FA and separated on a column (ReproSil-Pur C18-AQ, 1.9 μm, 75 μm inner diameter, 20 cm length; Dr Maisch) over a 78 min gradient (buffer A, 0.1% FA in water; buffer B, 0.1% FA in 80% ACN) at a flow rate of 300 nL/min. MS1 was analyzed with a scan range (m/z) of 350–1,550 at an Orbitrap resolution of 120,000. The RF lens, AGC target, maximum injection time, and exclusion duration were 30%, 1.0e6, 50 ms, and 15 s, respectively. MS2 was analyzed with an isolation window (m/z) of two at an Orbitrap resolution of 15,000. The AGC target, maximum injection time, and HCD type were 5.0e4, 80 ms, and 35%, respectively. For further verification, the same samples were analyzed using electron transfer/higher energy collision-induced dissociation (EThcD) in mass spectrometry (Orbitrap Fusion Lumos mass spectrometer). The MS1 was analyzed with a mass range of 400–1,600 at a resolution of 120,000 at 200 m/z. The RF Lens was set as 30% and the maximum injection time (MIT) was 100 ms. The MS2 was analyzed in quadrupole mode and the isolation window was 2 m/z. The EThcD collision energy type was 35%. The MIT was set at 250 ms and cycle time was set at 3 s.

### Data analysis

Raw data files were searched against the SARS-CoV-2 S protein sequence using Byonic™ software (version 3.6.0, Protein Metrics, Inc.), with the mass tolerance for precursors and fragment ions set at ±10 and ±20 ppm, respectively. HCD or EThcD was chosen as the fragmentation type. Two missed cleavage sites were subjected to trypsin or trypsin/Glu-C digestion. The fixed modification was carbamidomethyl (C), and the variable modifications included oxidation (M), acetyl (protein N-term), and de-amidation (N). In addition, the six most common *O*-glycans (HexNAc(1) with mass of 203.079 Da; HexNAc(2) with mass of 406.159 Da; HexNAc(1)Hex (1) with mass of 365.132 Da; HexNAc(2)Hex (1) with mass of 568.212 Da; HexNAc(1)Hex (1)NeuAc (1) with mass of 656.228 Da; and HexNAc(1)Hex (1)NeuAc (2) with mass of 947.323 Da) were specified as *O*-glycan modifications for intact *O*-glycopeptides. We then added the protein database and the decoy database. All other parameters were set to the default values, and protein groups were filtered using a 1% false discovery rate, based on the number of hits obtained for the searches against the databases. Stricter quality control methods for intact *O*-glycopeptide identification were implemented; they required a score of not less than 300, and at least six amino acids to be identified. Furthermore, all the glycopeptide-spectrum matches (GPSMs) were examined manually by checking the oxonium ions and b/y/c/z ions to ensure the correct identification of the glycopeptides and their glycan compositions, and distinguish the confident *O*-glycosites and their linked glycans from the uncertain glycosites within a specific glycopeptide. In addition, these *O*-glycosites had to be identified repeatedly at least twice. Model building based on the Cryo-EM structure (PDB: 6VSB) of the SARS-CoV-2 S protein was performed using PyMOL.

## Results and Discussion

### Strategy for Intact O-Glycopeptide Analysis

Our previous study, as well as others, have revealed site-specific *N-*glycosylation of recombinant S proteins (Watanabe et al., 2020; Zhang et al., 2020). Two or more potential *O*-glycosites have also been discovered using LC-MS/MS in recent studys (Shajahan et al., 2020b; Bagdonaite et al., 2021). However, comprehensive *O-*glycosylation analysis of the SARS-CoV-2 S protein has not been performed. In the present study, we aimed to characterize the *O-*glycosylation landscapes of SARS-CoV-2 recombinant S proteins by analysis of intact *O*-glycopeptides, including potential *O*-glycosites and their linked *O*-glycans.

**FIGURE 1:**
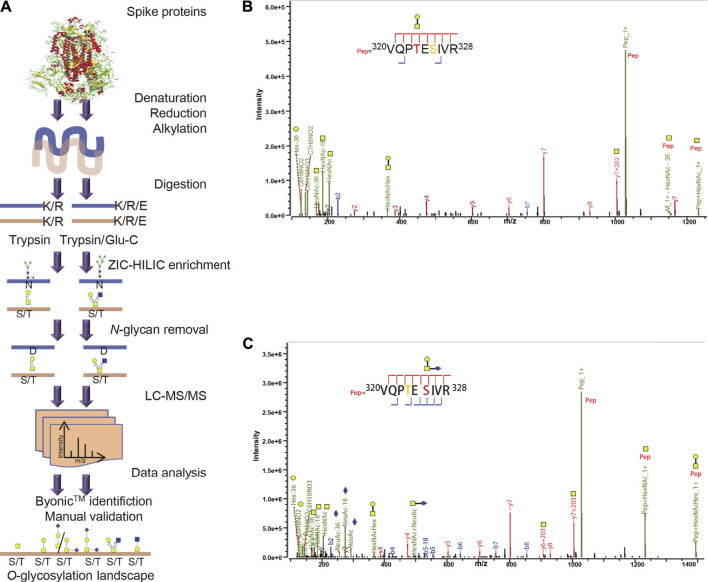
Comprehensive *O*-glycosylation profiling of SARS-CoV-2 spike proteins. **(A)** SARS-CoV-2 spike proteins expressed in insect or human cells were digested using trypsin or a mixture of trypsin and Glu-C. After ZIC-HILIC enrichment and PNGase F digestion, intact *O*-glycopeptides were analyzed using a high-resolution mass spectrometer, and their spectra were characterized using Byonic™ software and validated manually. **(B)** HCD-MS/MS spectrum of reported representative *O*-glycopeptide ^320^VQPTESIVR^328^ with deduced GalNAcGal glycan detected on site Thr323 or Ser325 of human spike protein subunit 1. **(C)** HCD-MS/MS spectrum of this *O*-glycopeptide with deduced GalNAcGalNeuAc glycan detected on site Thr323 or Ser325.

The strategy for intact *O*-glycopeptide analysis is shown in Figure 1A. The recombinant SARS-CoV-2 S proteins were digested using trypsin or a mixture of trypsin and Glu-C to cover as many potential *O*-glycosites as possible. Then, intact glycopeptides were enriched using ZIC-HILIC (Pohlentz et al., 2016), and de-*N-*glycosylated with PNGase F to avoid interference from non-glycopeptides and *N*-glycopeptides. Finally, intact *O*-glycopeptides were analyzed using a high-resolution mass spectrometer, and their mass spectra were characterized using Byonic™ software and validated manually (Zhang et al., 2018). It is worth remarking that *O*-glycosylation assignment to a specific amino acid by Byonic™ is not always confident when multiple Ser/Thr residues are present within the glycopeptide, especially when using HCD-type MS2 fragmentation. These *O*-glycosites were classified into the potential sites in this study.

The S protein expressed in insect cells contained 1,209 amino acids (residues 16–1,213), including 94 Thr and 92 Ser residues regarded as potential *O*-glycosites. The spike protein S1 subunit expressed in human cells contained 681 amino acids (residues 16–685), including 57 Thr and 50 Ser residues as potential *O*-glycosites (Supplementary Figure S1). Combined digestion strategy can improve glycosite identification and glycoprotein sequence coverage (Chen et al., 2011). To evaluate our method based on MS analysis and data analysis by Byonic in this study, we first analyzed the two previously reported O-glycosites, T323 and S325. The spike protein subunits S1 and S2 expressed on human cells were digested by trypsin and/or chymotrypsin, and analyzed by stepped HCD product triggered CID (HCD-pd-CID) without glycopeptide enrichment and PNGase F digestion (Shajahan et al., 2020b). As shown in Figure 1B, Byonic™ analysis disclosed the presence of the *O*-glycopeptide ^320^VQPTESIVR^328^ with an uncertain *O*-glycosite at T323 based on the b/y ions with or without glycan retention. It is worth noting that S325 was an alternative glycosite in this peptide because only the “∼y7+203” ion with glycan retention was detected, although Byonic™ tended to assign the T323 according to the y4/y5 ions without the linked glycan. However, the presence of ∼y6/∼y7/∼y8/∼b7 ions without the glycan indicates the fact that the b/y ions produced by HCD tends to lose their glycans (Pap et al., 2018). Without sufficient b/y ions with glycan retention, the confident *O*-glycosite cannot be determined in the glycopeptide. Shajahan et al. has reported that T323 seems a predominantly occupied site in an *O*-glycopepitde with the same peptide sequence ^320^VQPTESIVR^328^ through HCD fragmentation (Shajahan et al., 2020b), suggesting T323 is a high-probability glycosite. Similarly, the representative HCD-MS/MS spectra in Figure 1C revealed the presence of an uncertain *O*-glycosite at S325, which also could be T323 due to high frequency loss of entire glycan in HCD fragmentation. Hence, both T323 and S325 are uncertain *O*-glycosites and could not be confident identified by the HCD-MS/MS spectra in Figures 1B,C. These results indicate that our strategy is feasible for *O-*glycosylation profiling.

### Comprehensive O-Glycosylation Profiling of Recombinant SARS-CoV-2 S Protein Expressed in Insect Cells

**FIGURE 2:**
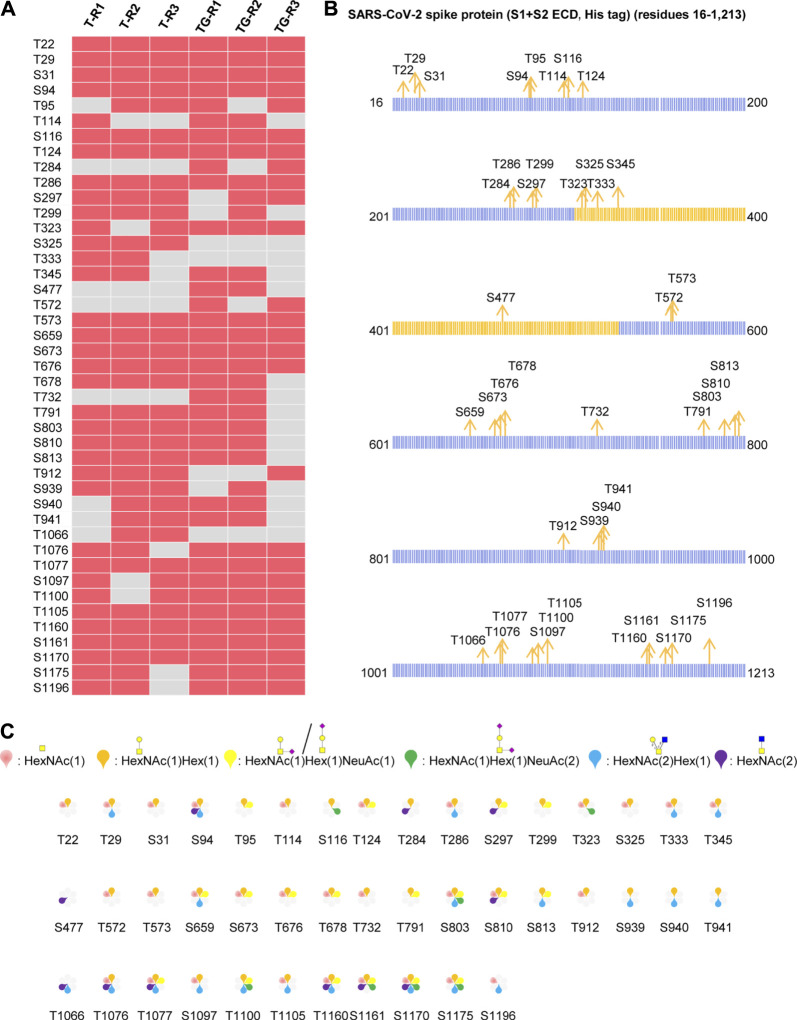
Comprehensive *O*-glycosylation characterization of recombinant SARS-CoV-2 S protein (S1+S2 ECD, His tag) expressed in insect cells. **(A)** Uncertain *O*-glycosites identified using trypsin (T) or typsin/Glu-C (TG) in three replicates. **(B)** Mapping of identified *O*-glycosites to amino acid sequences. RBD is highlighted in yellow. **(C)** *O*-glycan compositions on each site.

The S protein produced by the baculovirus insect cell expression system contained 186 potential *O*-glycosites. Using our aforementioned strategy, a total of 255 intact O-glycopeptides composed of 50 peptides backbones and 43 uncertain *O*-glycosites were discovered by HCD (Supplementary Table S1 and Figure S2). In these glycopeptides, 40 potential *O*-glycosites, except S477, T572, and T732 were found repeatedly using trypsin alone. Using trypsin combined with Glu-C, three more *O*-glycosites were discovered, although another three *O*-glycosites (S325, T333, and T1066) were missed due to combinational digestion (Figure 2A). Hence, although trypsin digestion can yield good identification results, trypsin combined with Glu-C digestion should be considered as complementary step because that some suitable glycopeptides can be easily detected by mass spectrometry. Furthermore, we mapped these *O*-glycosites to the amino sequences, and found that the *O*-glycosites appeared in several areas, especially in the N- and C-termini of the S protein (Figure 2B). It is notable that the *O*-glycosites T323, S325, T333, S345, and S477 were located in the receptor-binding domain (RBD). These results indicate that the SARS-CoV-2 S protein is an *O*-glycoprotein with a large number of *O-*glycosites. In addition, the number of *O-*glycosylated Thr residues 25) was higher than that of *O-*glycosylated Ser residues 18) (Figure 2B). This result is consistent with those of previous studies on *O-*glycosites (Zhang et al., 2018). Finally, a global *O-*glycan composition analysis was performed (Figure 2C). Six *O*-glycan compositions were identified on these sites, including HexNAc(1), HexNAc(2), HexNAc(1)Hex (1), HexNAc(2)Hex (1), HexNAc(1)Hex (1)NeuAc (1), and HexNAc(1)Hex (1)NeuAc (2). Regarding the frequency of these glycans on different glycosites, occupancies with HexNAc(1)Hex (1), HexNAc(1), HexNAc(2)Hex (1), HexNAc(1)Hex (1)NeuAc (1), HexNAc(2), and HexNAc(1)Hex (1)NeuAc (2) compositions were found on 40, 30, 21, 18, 11, and seven glycosites, respectively. Moreover, most glycosites contained at least two types of *O*-glycans, a majority of which were non-sialylated (Figure 2C). It’s worth noting that NeuAc would rarely found on insect *O*-glycans, because that insect cells generally lack adequate levels of the glycosyltransferases to synthesize sialylated products, especially if there’s not NeuAc oxonium ion in the MS2 spectrum (Figure 2C). When that happens, the spectrum may be a half-right identification (right peptide with wrong *O*-glycans) although with high Byonic score. These results indicate the *O*-glycans appeared on the recombinant SARS-CoV-2 S protein expressed in insect cells.

### Comprehensive O-Glycosylation Profiling of Recombinant SARS-CoV-2 S Protein Expressed in Human Cells

**FIGURE 3:**
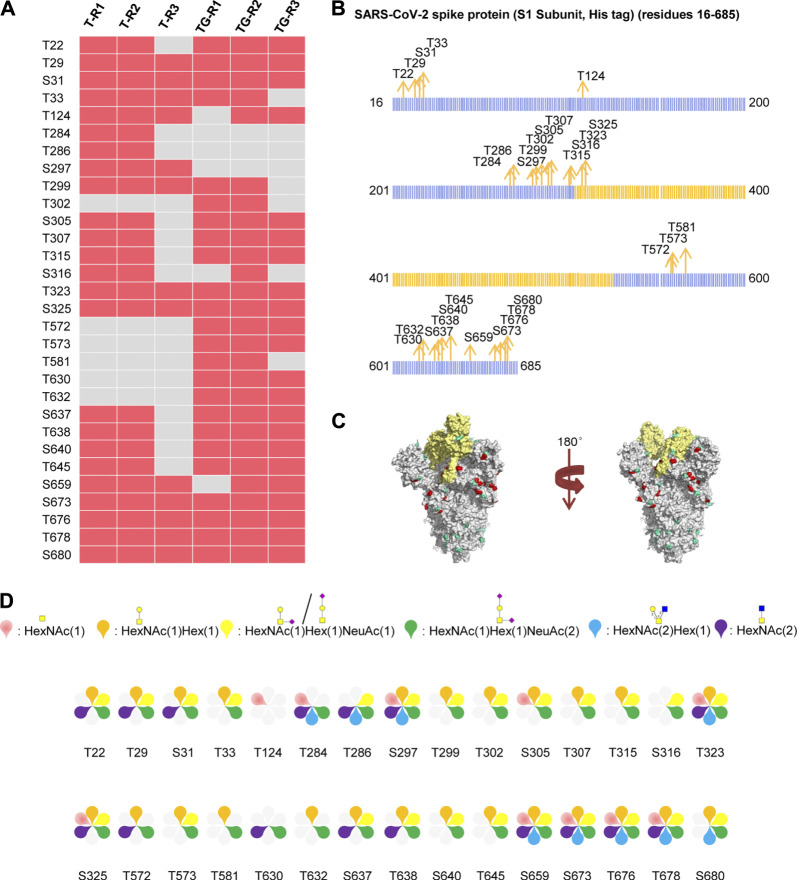
Comprehensive *O*-glycosylation characterization of SARS-CoV-2 S protein (S1, His tag) expressed in human cells. **(A)** Uncertain *O*-glycosites identified using trypsin (T) or typsin/Glu-C (TG) in three replicates. **(B)** Mapping of identified *O*-glycosites to amino acid sequences. RBD is highlighted in yellow. **(C)** *O*-glycosites (red) and *N*-glycosites (blue) in three-dimensional structure of SARS-CoV-2 S protein trimers (PDB code: 6VSB). RBD is highlighted in yellow. **(D)** *O*-glycan compositions on each site.

The recombinant SARS-CoV-2 S protein S1 subunit produced by the human cell expression system was used for analysis of the *O*-glycans, as the *O*-glycan compositions in insect cells could be different from those in human cells. Using our aforementioned strategy, 407 intact *O*-glycopeptides composed of 34 peptides backbones and 30 uncertain *O*-glycosites (20 Thr and 10 Ser residues) were discovered by HCD (Supplementary Table S2 and Figure S3). 24 and 27 uncertain *O*-glycosites were found repeatedly using trypsin and a mixture of trypsin/Glu-C, respectively. The trypsin combined with Glu-C digestion can increase the number of identified *O*-glycosites (Figure 3A). The results showed that the two digestion methods were complementary for *O*-glycosite identification. Furthermore, we mapped these 30 *O*-glycosites to the amino sequences. We found that the *O*-glycosites mainly appeared at the S1 subunit and RBD (Figures 3B,C). It is notable that two conserved *O*-glycosites, T323 and S325, were located in the RBD of the S1 subunit, and may play a critical role in viral binding with hACE2 receptors (Andersen et al., 2020; Hoffmann et al., 2020). A global *O-*glycan composition analysis of the S1 subunit was performed. *O*-glycan occupancies with HexNAc(1)Hex (1)NeuAc (2), HexNAc(1)Hex (1), HexNAc(1)Hex (1)NeuAc (1), HexNAc(2), HexNAc(1), and HexNAc(2)Hex (1) were found on 29, 25, 24, 16, 10, and nine glycosites, respectively. *O*-glycans on most glycosites were sialylated (Figure 3D). These results indicate the more complex *O-*glycosylation and the heterogeneity of *O*-glycan compositions on the recombinant SARS-CoV-2 S protein expressed in human cells.

### O-Glycosylation Landscapes of S1 Subunits Expressed in Insect and Human Cells

**FIGURE 4:**
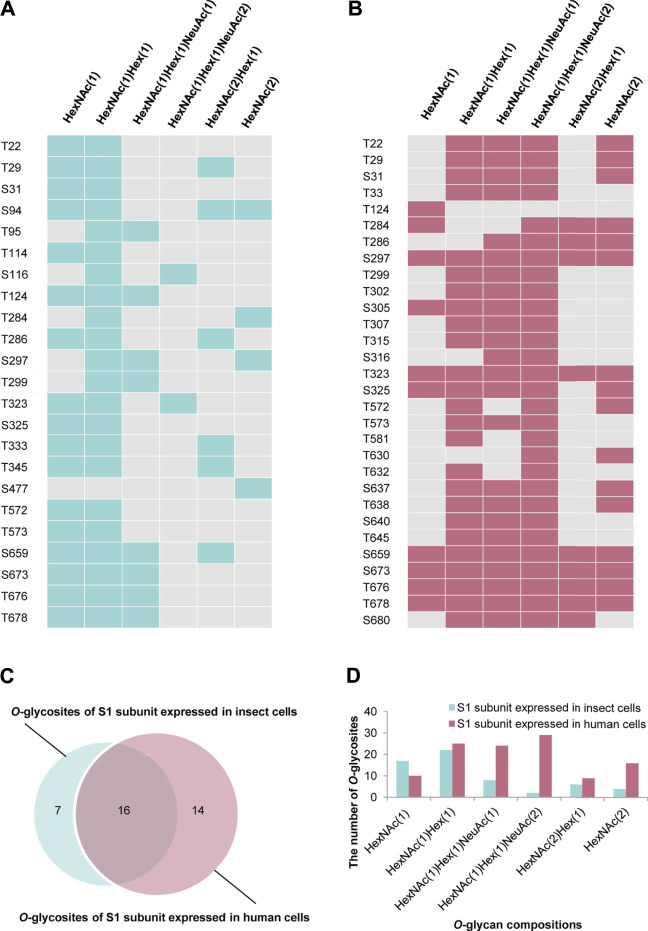
Comparison of *O*-glycosylation modifications of S1 subunits expressed in insect or human cells. **(A)** *O*-glycan compositions in each glycosite of S1 subunit expressed in insect cells. **(B)** *O*-glycan compositions in each glycosite of S1 subunit expressed in human cells. **(C)** Comparison of *O*-glycosites of S1 subunits expressed in different expression systems. **(D)** Number of S1 subunit *O*-glycosites attached by each type of *O*-glycan composition.

Based on the above findings, we further compared the *O-*glycosylation landscapes of the S1 subunits expressed in insect and human cells. 23 uncertain *O*-glycosites were present in the S1 subunit expressed in insect cells (Figure 4A). In contrast, 30 *O*-glycosites were present in the S1 subunit expressed in human cells (Figure 4B). In addition, 16 common *O*-glycosites (T22, T29, S31, T124, T284, T286, S297, T299, T323, S325, T572, T573, S659, S673, T676, and T678) were discovered in the S1 subunits expressed in both insect and human cells, including the two sites, T323 and S325, located in the RBD. Seven and 14 unique *O*-glycosites were found in the insect and human cell–produced S1 subunits, respectively (Figure 4C). It’s worth noting that HCD can identify the intact *O*-glycopeptide confidently while usually failing to distinguish a specific *O*-glycosite from multiple uncertain glycosites within a glycopeptide. More validation experiments were needed to compare the differences between the two S proteins. Furthermore, the number of S1 subunit *O*-glycosites occupied by each type of *O*-glycan compositions was very different. Most *O*-glycosites of the insect cell–produced S1 subunit contained HexNAc(1)Hex (1) and HexNAc(1). On the other hand, most *O*-glycosites of the human cell–produced S1 subunit contained HexNAc(1)Hex (1)NeuAc (2), HexNAc(1)Hex (1)NeuAc (1), and HexNAc(1)Hex (1) (Figure 4D). These results implied that the *O*-glycosite and *O*-glycan compositions varied with the host cell type, which could be taken into account when using the recombinant proteins for vaccine and drug development.

### Identification of Intact O-Glycopeptides Using EThcD

**FIGURE 5:**
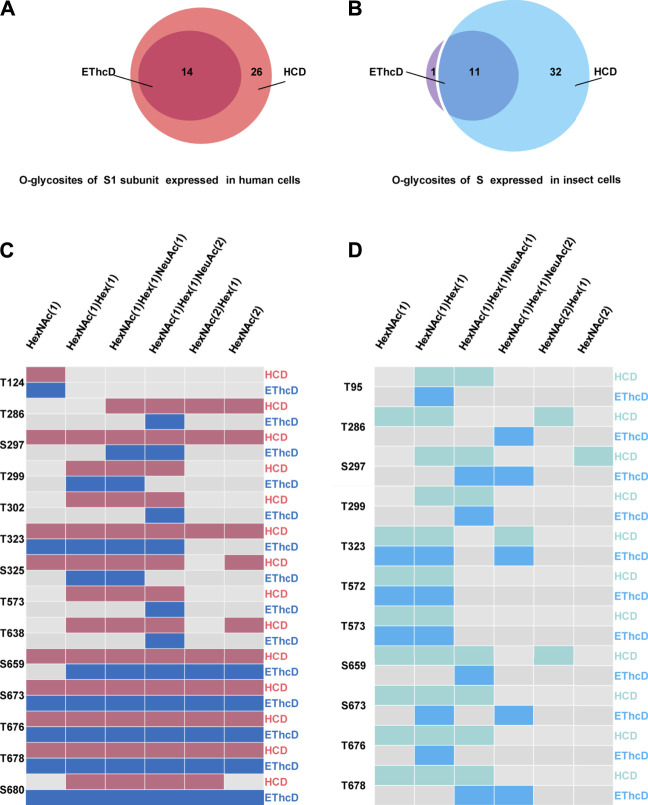
Systems analysis of O-glycosylation of spike protein via EThcD and HCD mass spectrometry. **(A)** Identified *O*-glycosites of S1 subunit expressed in human cells using HCD and EThcD. **(B)** Identified *O*-glycosites of S expressed in insect cells using HCD and EThcD. **(C)** The same *O*-glycosites of S1 subunit expressed in human cells identified using both HCD and EThcD. **(D)** The same *O*-glycosites of S expressed in insect cells identified using both HCD and EThcD.

Electron transfer dissociation (ETD) can produce extensive fragmentation of the peptide backbone, enabling sequencing of the peptide, while preserving glycans on the peptide backbone (Hogan et al., 2005; Myers et al., 2013). However, ETD frequently leads to incomplete fragmentation and massive residual precursor ions, and is usually combined with HCD or CID (Kolbowski et al., 2017). Our previous research had shown that EThcD can provide a more complete fragmentation of *O*-glycopeptides than HCD or ETD alone (Frese et al., 2013), leading to better *O*-glycosylation site localization (Zhang et al., 2018). Hence, all the above samples were reanalyzed in triplicate *via* EThcD. A total of 12 *O*-glycosites of S expressed in insect cells and 14 *O*-glycosites of S1 subunit expressed in human cells were assigned (Supplementary Table S3, Figure S4, S5). Compared with HCD identification results, EThcD identified less *O*-glycosites. 14 out of 30 *O*-glycosites identified *via* HCD on S1 subunit expressed in human cells were identified by EThcD (Figure 5A). By further checking c/z ions with glycan retention, 11 *O*-glycosites (T124, T302, T323, S325, T573, T638, S659, T678, S673, T676, S680) were assigned confidently (Supplementary Figure S5). Similarly, EThcD enabled identification of 12 *O*-glycosites on S protein produced in insect cells, in which only one *O*-glycosite S680 was not found by HCD (Figure 5B). By further checking c/z ions which contain *O*-glycans, three *O*-glycosites (T95, T323, T573) can be assigned confidently (Supplementary Figure S4). Furthermore, the *O*-glycan types attached to each *O*-glycosite were analyzed and many *O*-glycans identified by HCD, especially the more complex *O*-glycans such as HexNAc(1)Hex (1)NeuAc (2), were verified by EThcD since ETD preferentially retained the intact glycan moities (Figure 5C and Figure 5D). In addition, HexNAc(2)Hex (1) and HexNAc(2) were not verified by EThcD in the S expressed in insect cells, which suggested that the two *O*-glycans might be misidentified by HCD due to fragmentation of glycans. All of these results indicate that with both HCD and EThcD, we can identify a large number of intact *O*-glycopeptides of spike proteins. However, more verification experiments were still needed to identify more confident *O*-glycosites. These results proved that the SARS-CoV-2 S protein is a glycoprotein decorated with various *O*-glycans.

Recently, there are a few *O*-glycosites of S protein that are consistently identified by different groups. For example, Bagdonaite *et al.* used an *O*-glycoproteomic workflow based on in-gel digestion, de-N-glycosylation and desialylation strategy to map *O*-glycosites on S protein expressed in insect cell or human cell, and in total 25 *O*-glycosites were identified (Bagdonaite et al., 2021). There are some differences on the *O*-glycosites reported between their work and our reports. The reasons include using different expression cell strain, the same recombinant S protein from the same cell strain but cultured and processed in different labs or vendors, different sample preparation procedures, different mass spectrometer or analytical method, different software to process the data, even different identification criteria and threshold. They may lead to significant variations in glycosylation analysis. Even so, these methods and data may be useful for the development of vaccines and targeted drugs.

## Conclusions

In this study, we profiled a comprehensive *O-*glycosylation pattern of SARS-CoV-2 S proteins using optimized experimental procedure and HCD and EThcD mass spectrometry. There are 255 intact *O*-glycopeptides composed of 50 peptides sequences and 43 uncertain *O*-glycosites were discovered by HCD in insect cell–expressed S protein, and most of them were non-sialylated. There are three *O*-glycosites were confidently identified by EThcD. In contrast, in human S protein, 407 intact *O*-glycopeptides composed of 34 peptides sequences and 30 uncertain *O*-glycosites were discovered by HCD, 11 *O*-glycosites were unambiguously assigned by EThcD, and most of them were sialylated. However, the measurement of O-glycosylation occupancy hasn’t been made. Our results revealed that the SARS-CoV-2 S protein was modified by *O*-glycans, and that the *O*-glycosite and *O*-glycan compositions varied with the host cell type.

## Data Availability

The datasets presented in this study can be found in online repositories. The names of the repository/repositories and accession number(s) can be found in the article/Supplementary Material.
