# Supplementary material for: O-Glycosylation Landscapes of SARS-CoV-2 Spike Proteins
Source: Front Chem. 2021 Sep 6;9:689521. doi: 10.3389/fchem.2021.689521 (PMC8450404; doi:10.3389/fchem.2021.689521)
Supplement: Supplementary file 4 [file DataSheet1.DOCX]

**Supporting information for**

O-glycosylation Landscapes of SARS-CoV-2 Spike Proteins

Yong Zhang^1*^, Wanjun Zhao^2^, Yonghong Mao^3^, Yaohui Chen^3^, Shanshan Zheng^1^, Wei Cao^1^, Jingqiang Zhu^2^, Liqiang Hu^1^, Meng Gong^1^, Jingqiu Cheng^1*^, Hao Yang^1*^

^1^Key Laboratory of Transplant Engineering and Immunology, MOH; West China-Washington Mitochondria and Metabolism Research Center; Frontiers Science Center for Disease-related Molecular Network, West China Hospital, Sichuan University, Chengdu 610041, China.

^2^Department of Thyroid Surgery, West China Hospital, Sichuan University, Chengdu 610041, China.

^3^Institute of Thoracic Oncology, West China Hospital, Sichuan University, Chengdu 610041, China.

*To whom correspondence should be addressed: yanghao@scu.edu.cn, jqcheng@scu.edu.cn and nankai1989@foxmail.com

**Supplementary Figures:**

**Supplementary Figure S1.** Potential *O*-glycosites of SARS-CoV-2 S proteins expressed in insect and human cells

**Supplementary Figure S2.** Spectra of intact *O*-glycopeptides of SARS-CoV-2 S protein expressed in insect cells with ambiguously assigned *O*-glycosites by HCD

**Supplementary Figure S3.** Spectra of intact *O*-glycopeptides of SARS-CoV-2 S protein expressed in human cells with ambiguously assigned *O*-glycosites by HCD

**Supplementary Figure S4.** Spectra of intact *O*-glycopeptides of SARS-CoV-2 S protein expressed in insect cells with ambiguously and unambiguously assigned *O*-glycosites by EThcD

**Supplementary Figure S5.** Spectra of intact *O*-glycopeptides of SARS-CoV-2 S protein expressed in human cells with ambiguously and unambiguously assigned *O*-glycosites by EThcD

**Supplementary Table S1.** Intact *O*-glycopeptides of SARS-CoV-2 spike protein expressed in insect cells

**Supplementary Table S2.** Intact *O*-glycopeptides of SARS-CoV-2 spike protein expressed in human cells

**Supplementary Table S3.** Identified intact *O*-glycopeptides by EThcD

**Supplementary Figure S1.** Potential *O*-glycosites of SARS-CoV-2 S proteins expressed in insect and human cells


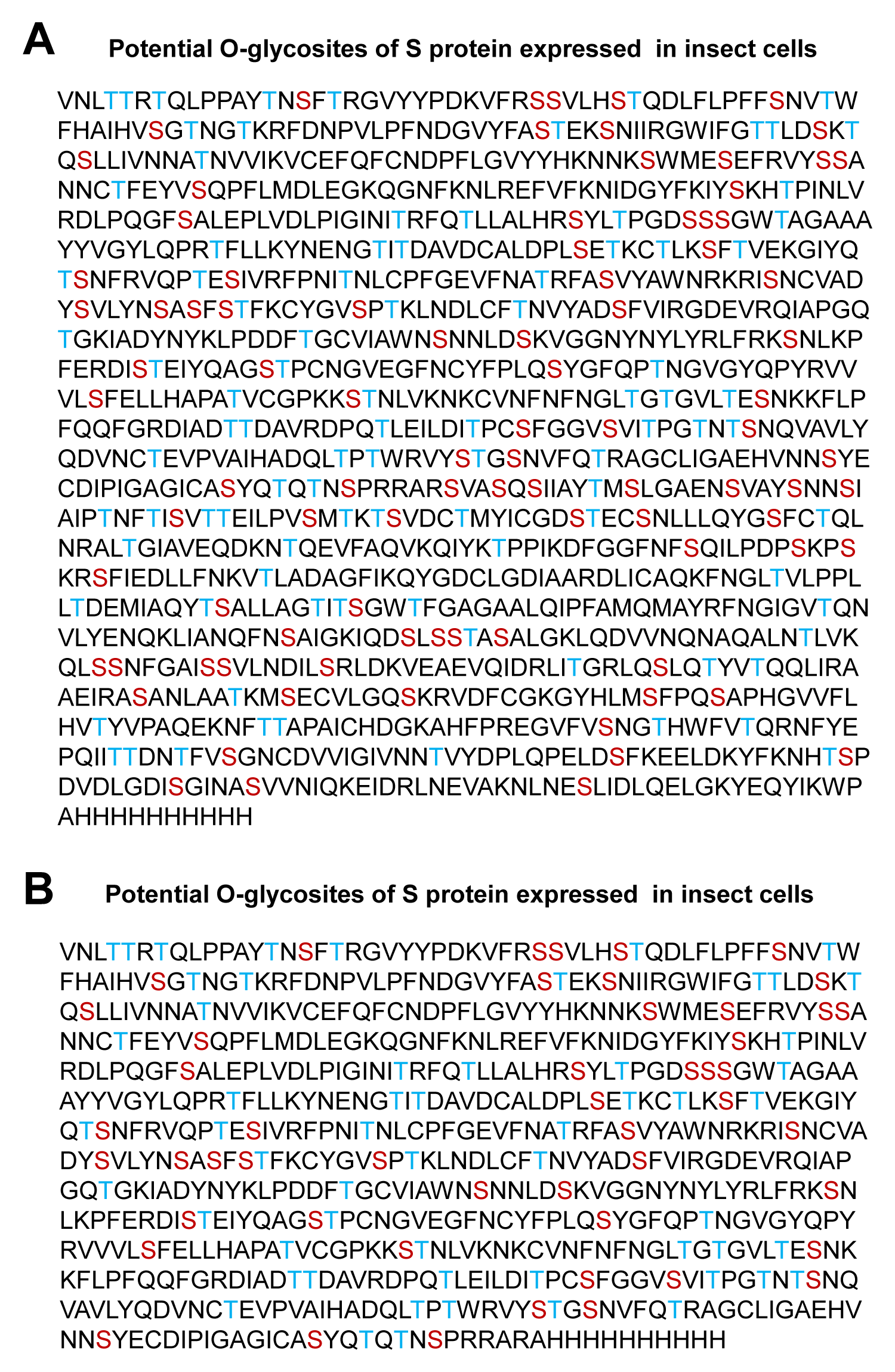


**human**

**Supplementary Figure S2.** Spectra of intact *O*-glycopeptides of SARS-CoV-2 S protein expressed in insect cells with ambiguously assigned *O*-glycosites by HCD

**T22 & S31**


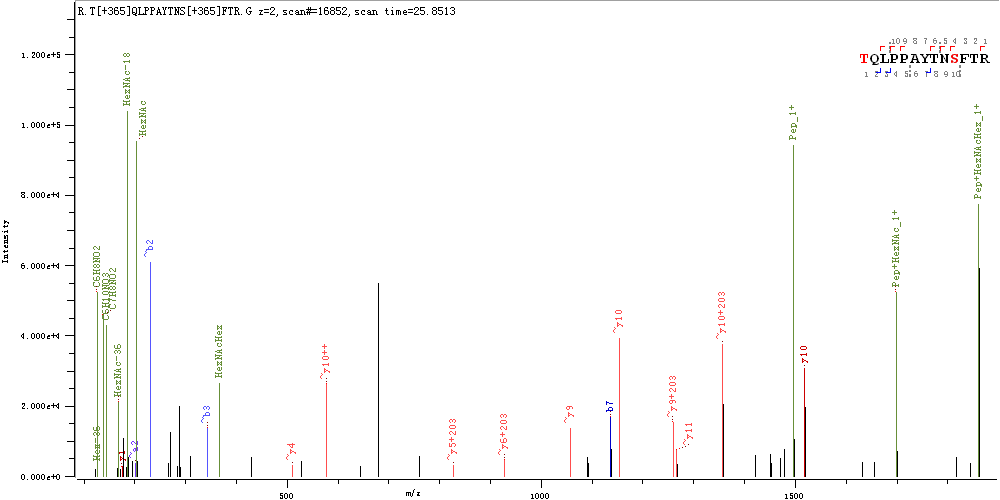


**T29**

**
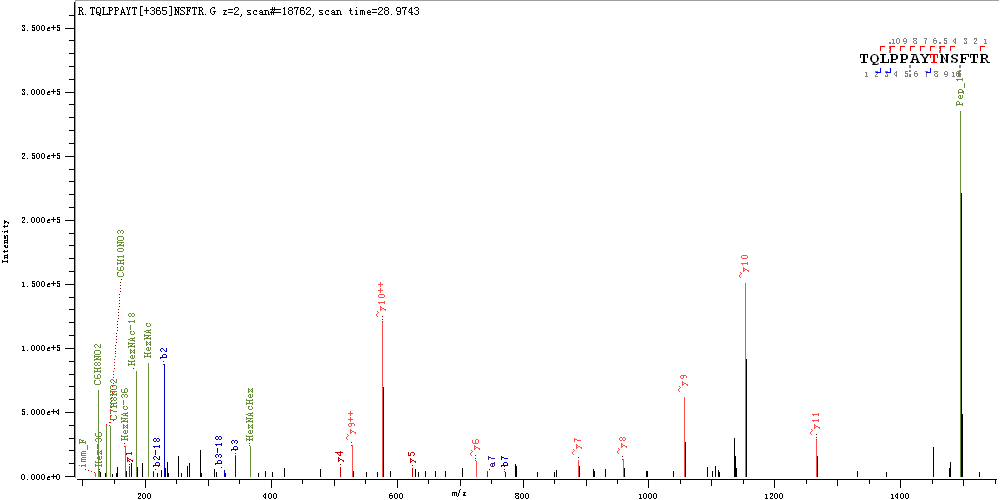
**

**S94**

**
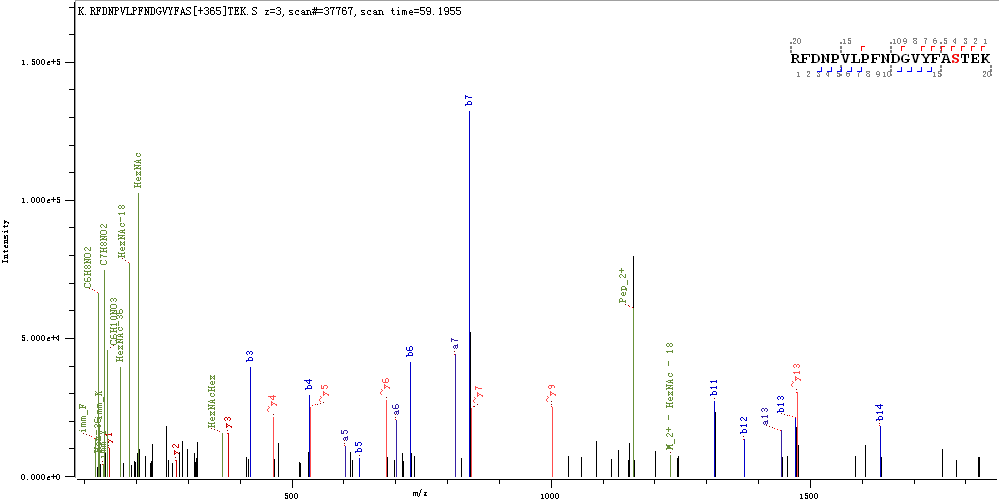
**

**S94 & T95**


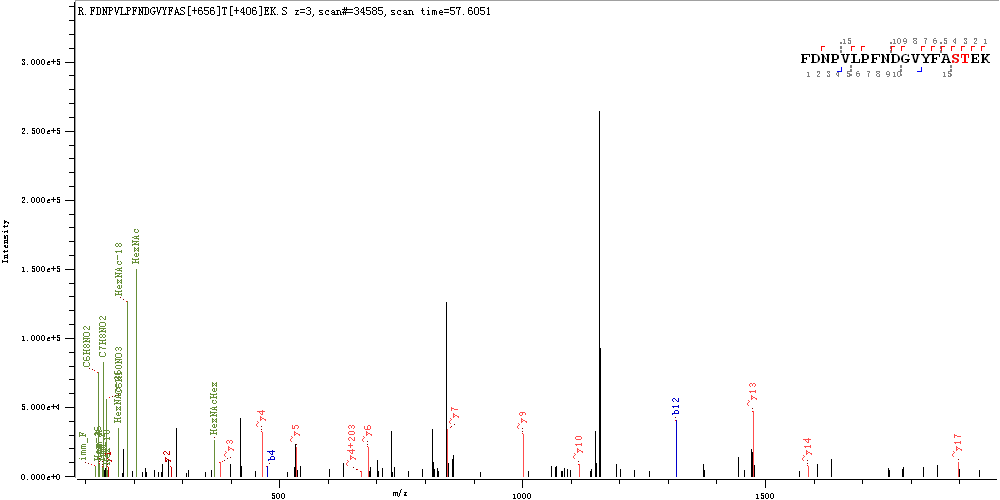


**T114 & S116 &T124**


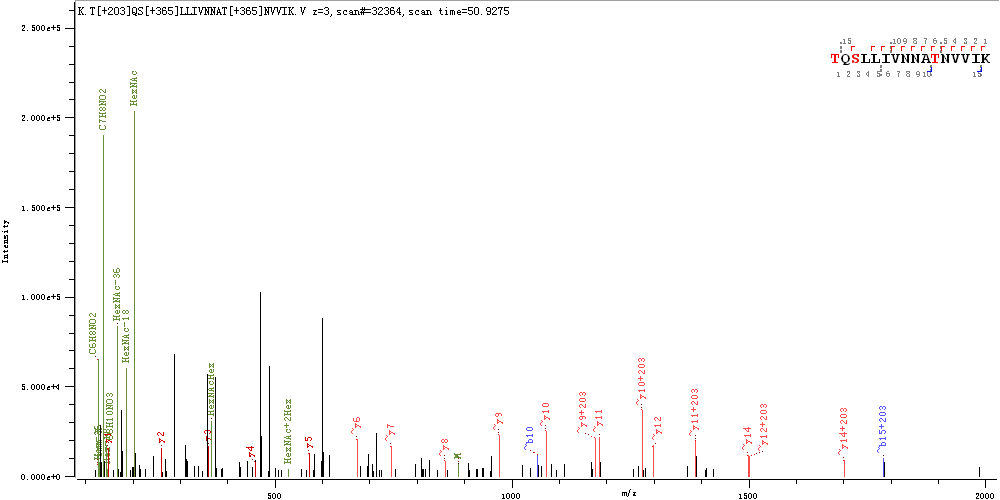


**T284 & T286 & S297**


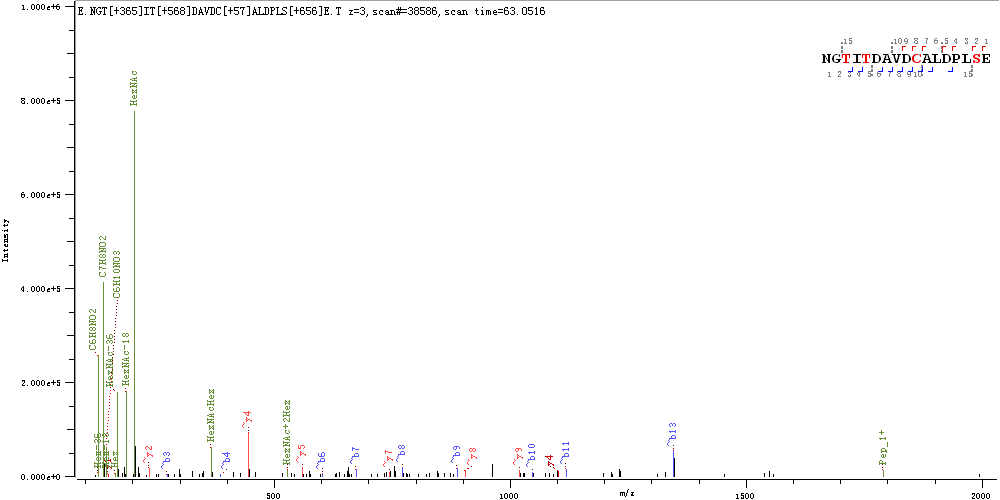


**T286 & S297&T299**


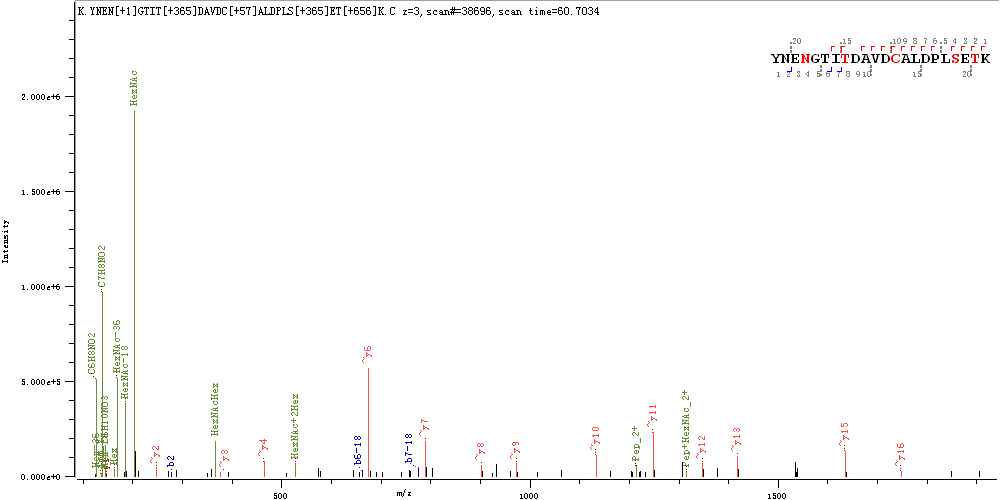


**T323**


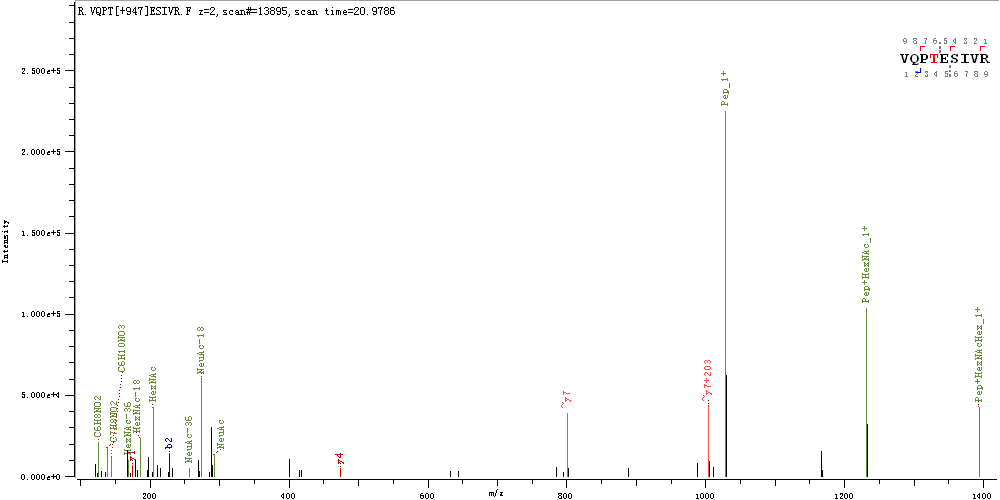


**S325**


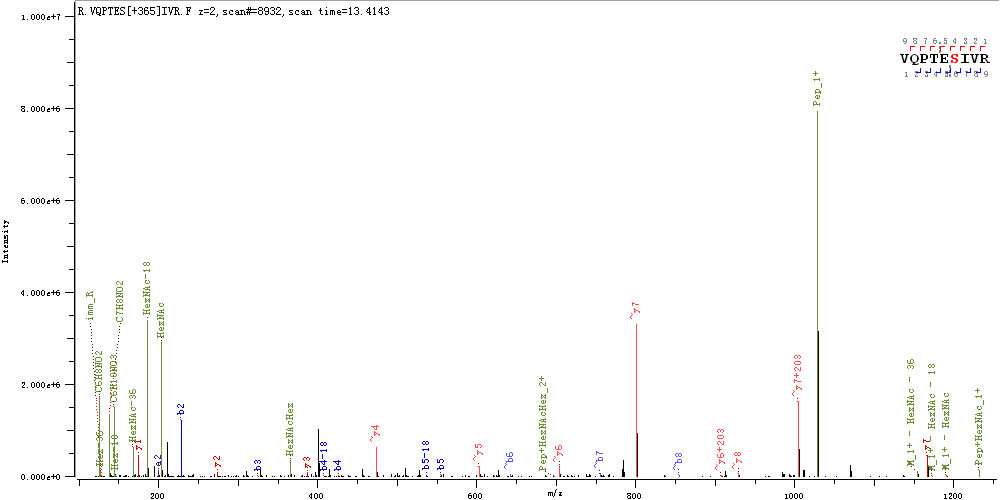


**T333 & T345**

**
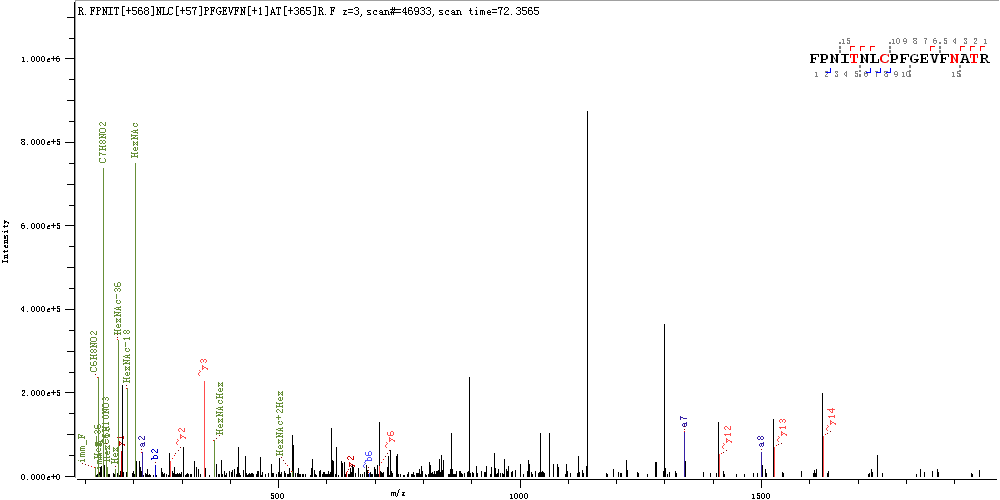
**

**S477**

**
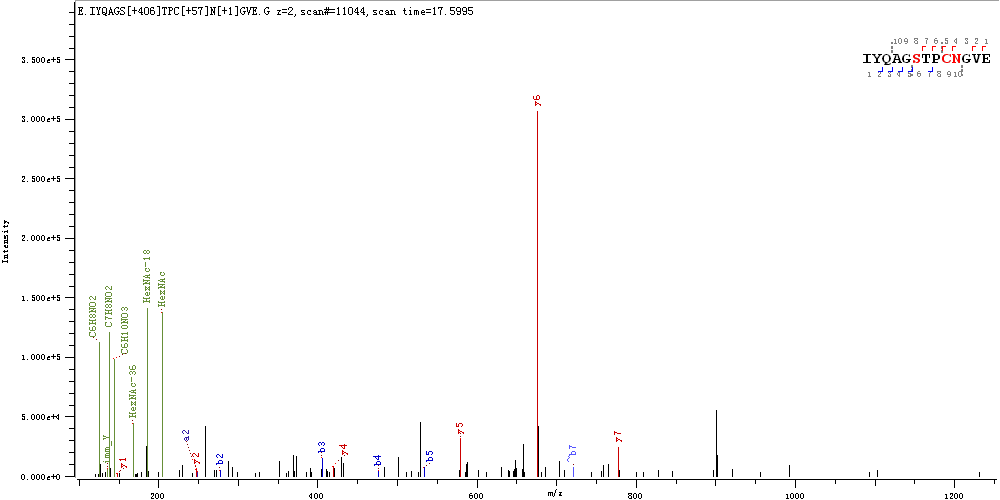
**

**T572**

**
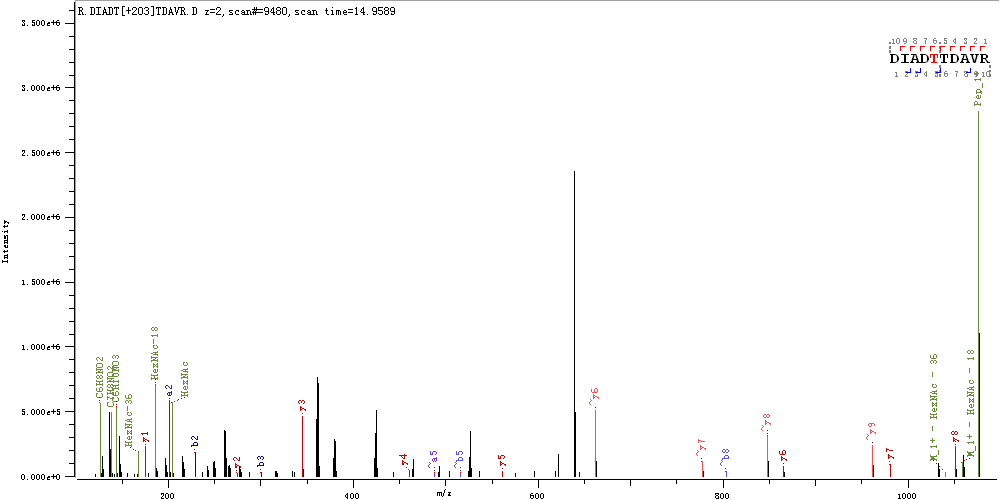
**

**T573**

**
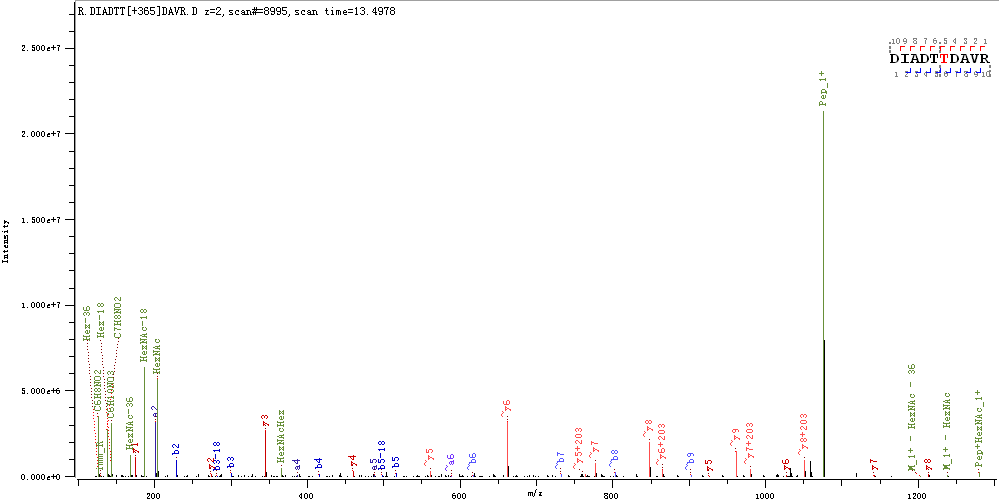
**

**S659 & T676 & T678**

**
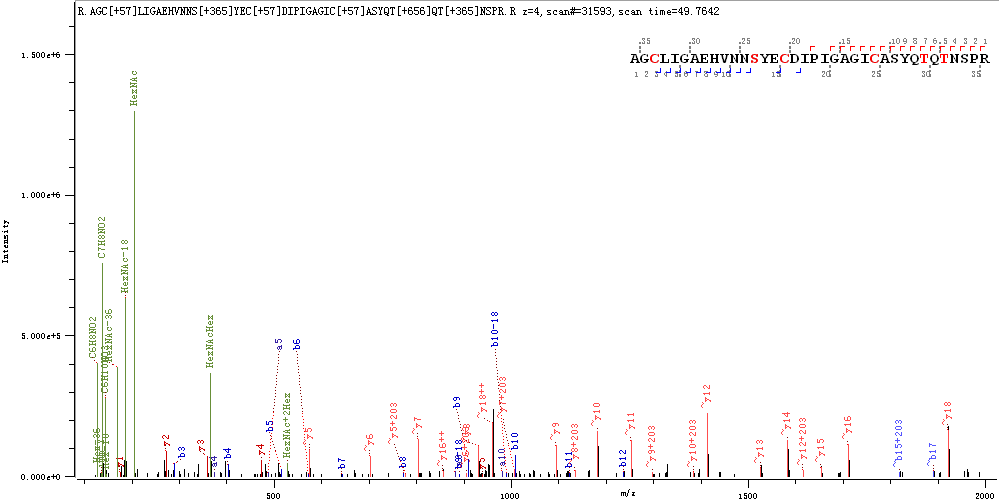
**

**S659 & S673**

**
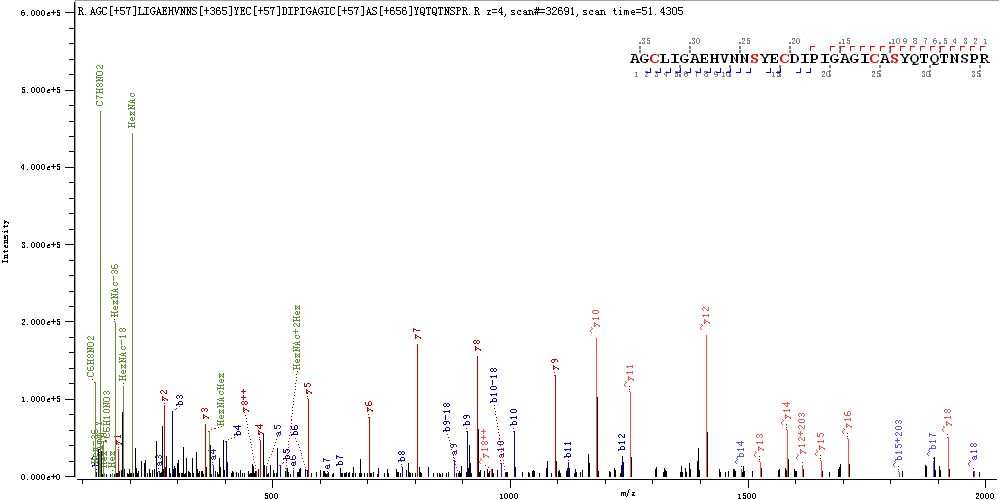
**

**T732**

**
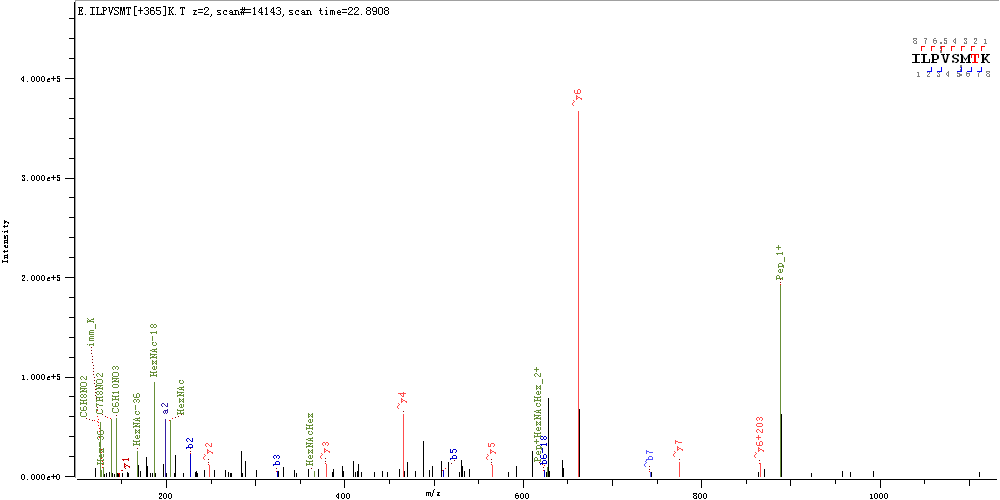
**

**T791 & S803 & S813**

**
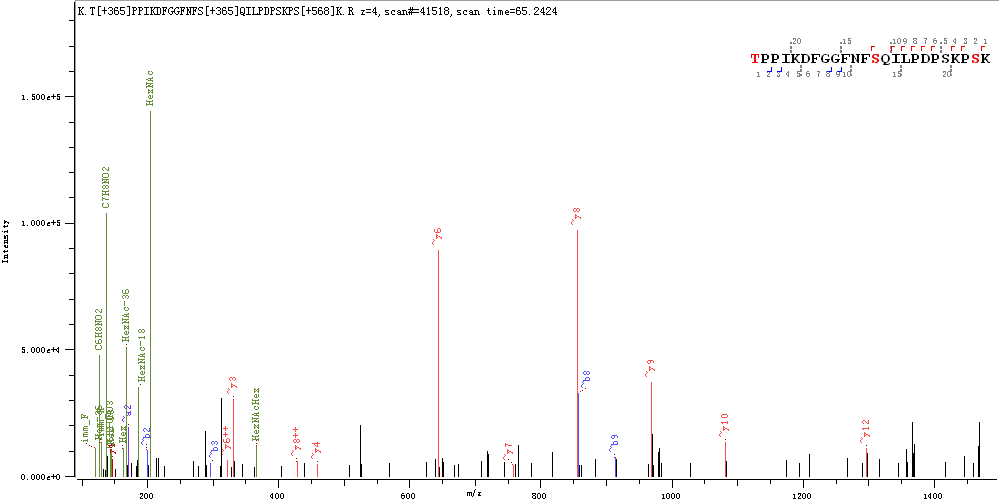
**

**S810 & S813**

**
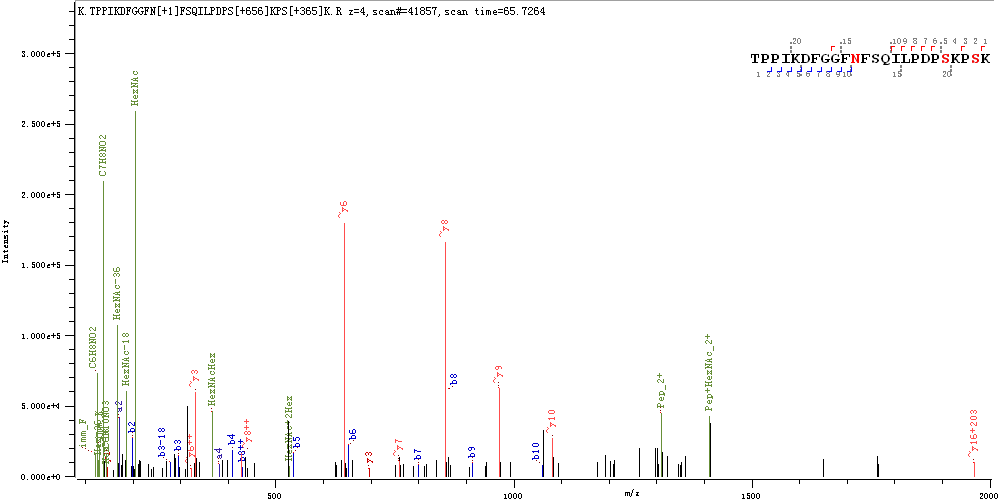
**

**T912**

**
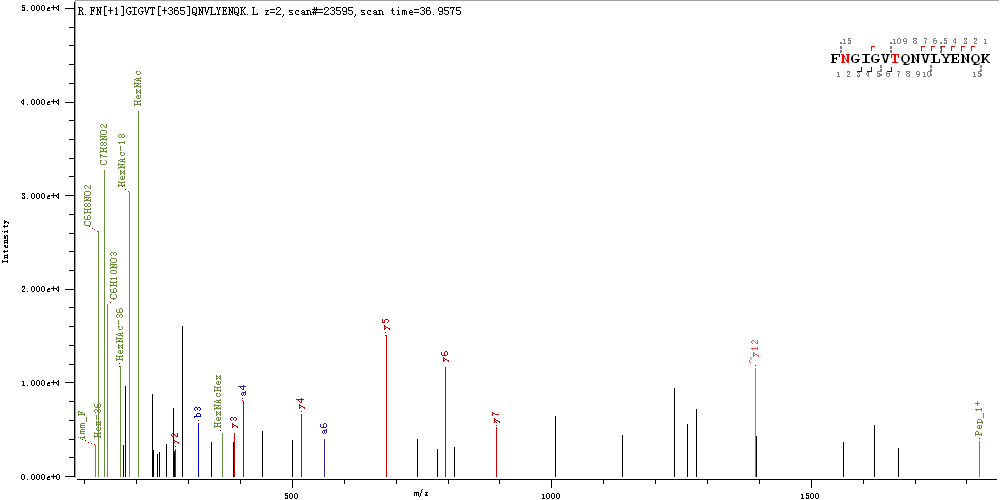
**

**S939**

**
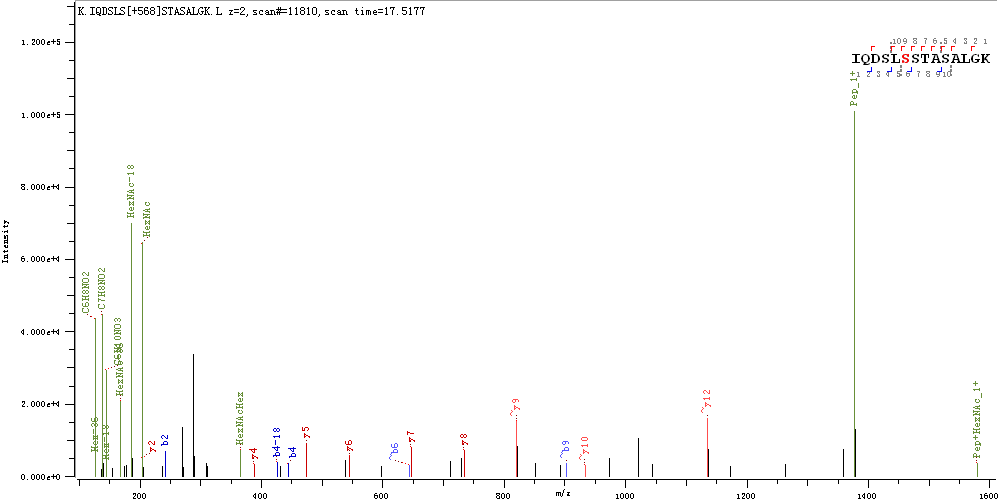
**

**S940 & T941**

**
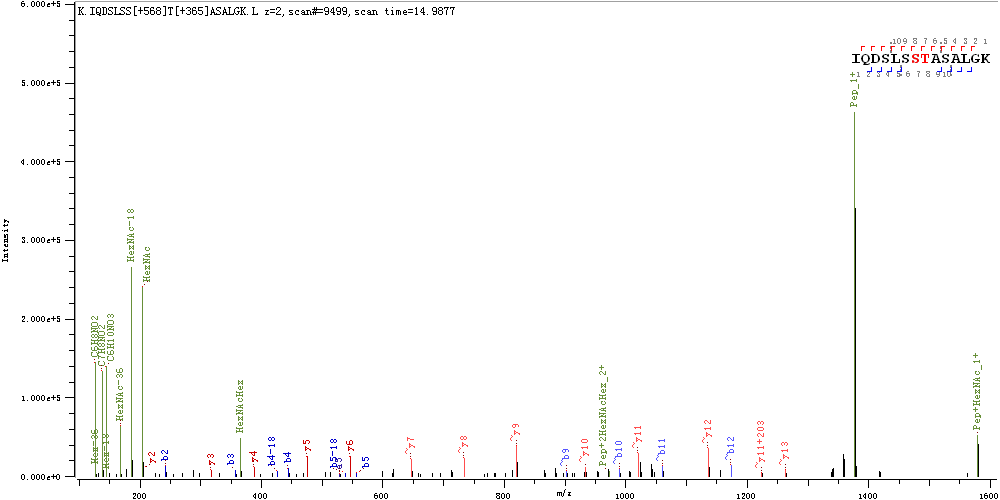
**

**T1066**

**
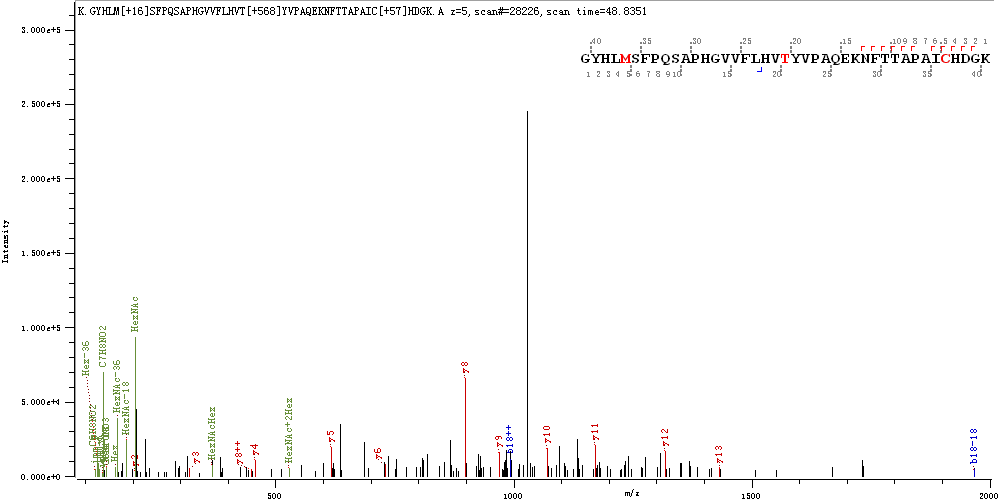
**

**T1076 & T1077**

**
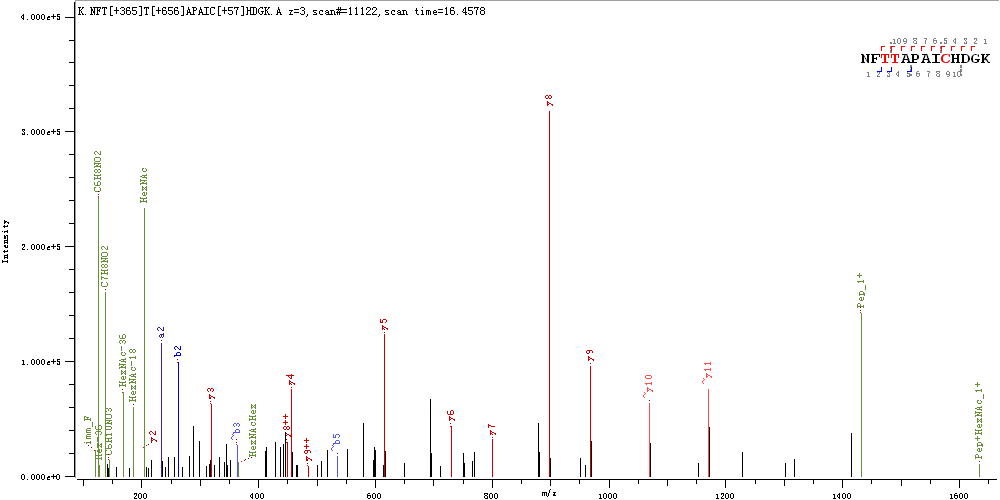
**

**S1097 & T1100**

**
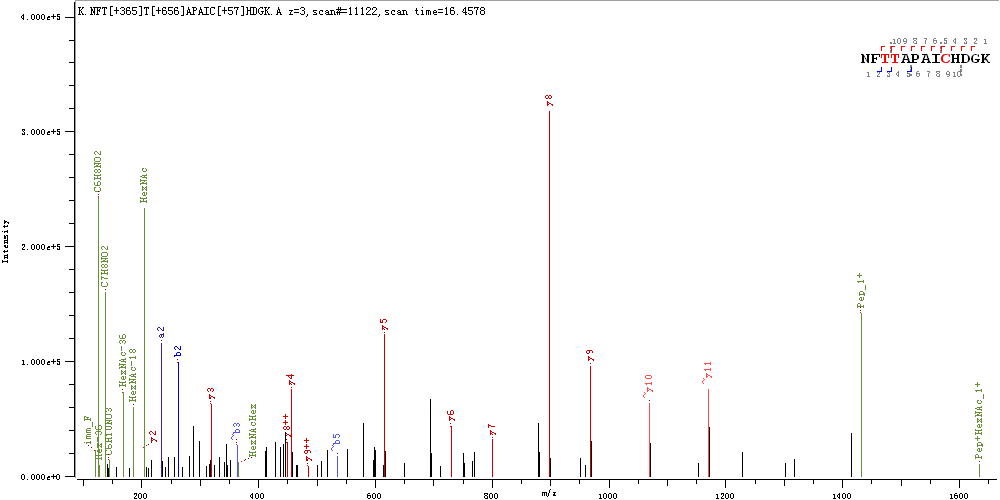
**

**T1105**

**
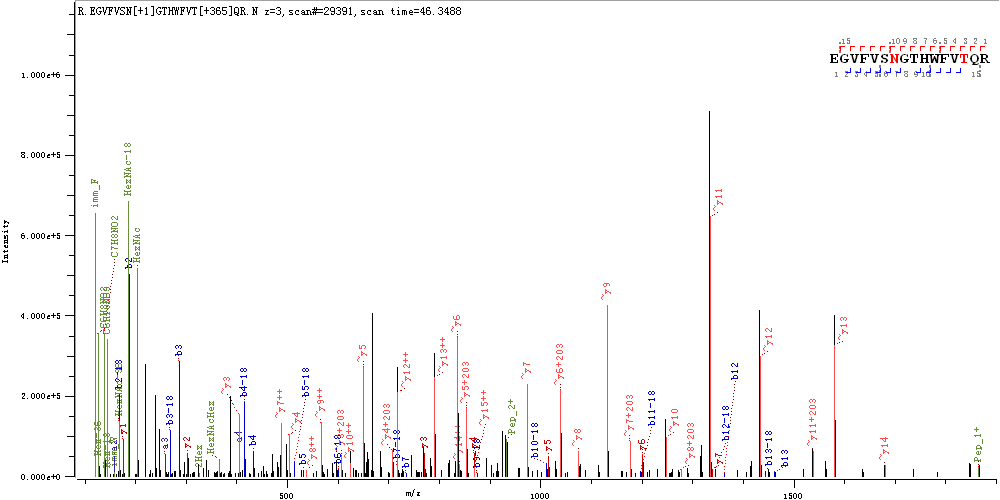
**

**T1160 & S1170**

**
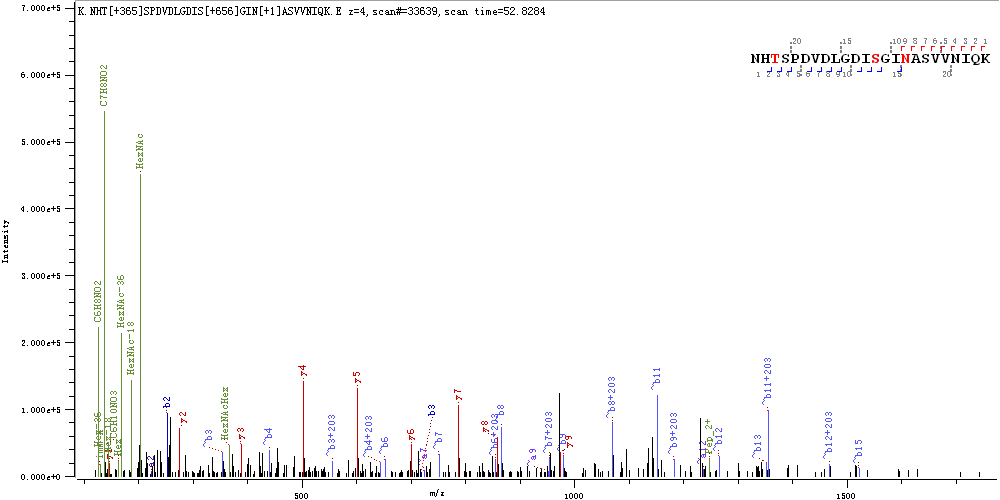
**

**S1161 & S1170**

**
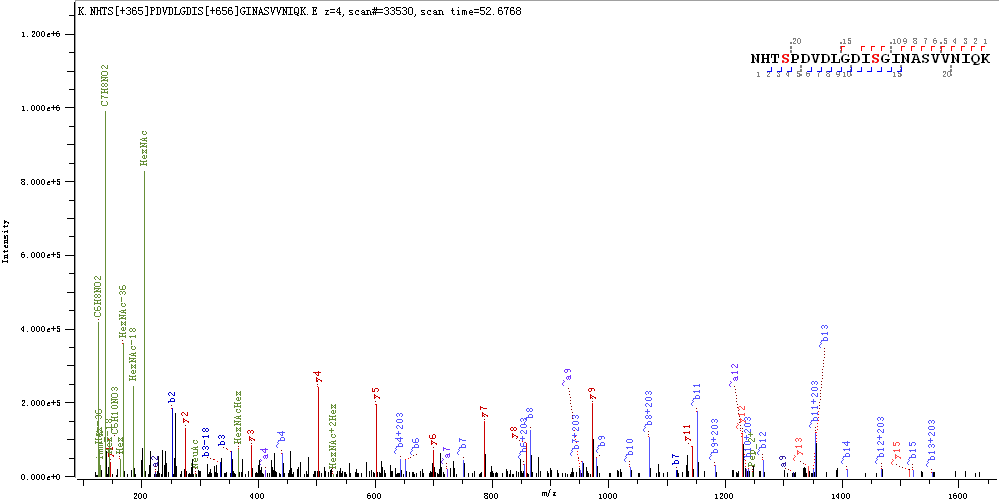
**

**S1170 & S1175**

**
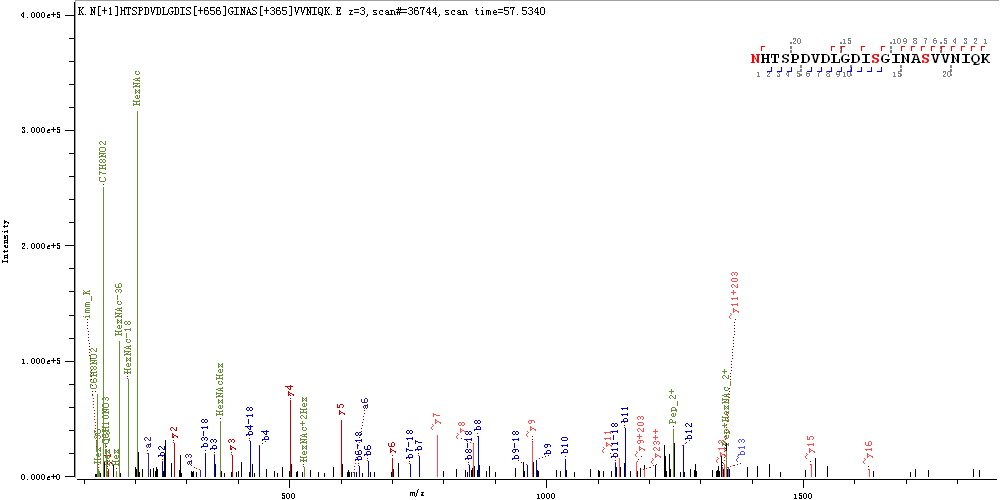
**

**S1196**

**
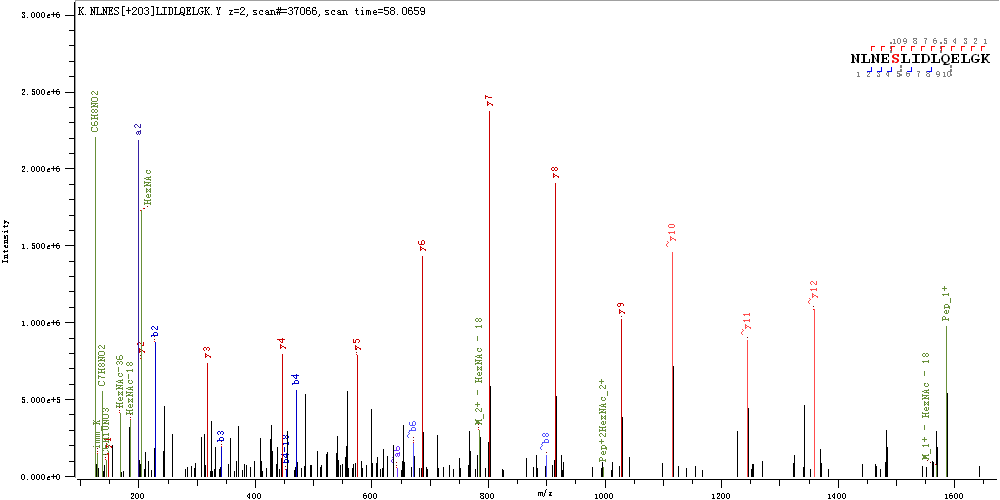
**

**Supplementary Figure S3.** Spectra of intact *O*-glycopeptides of SARS-CoV-2 S protein expressed in human cells with ambiguously assigned *O*-glycosites by HCD

**T22 & T29**


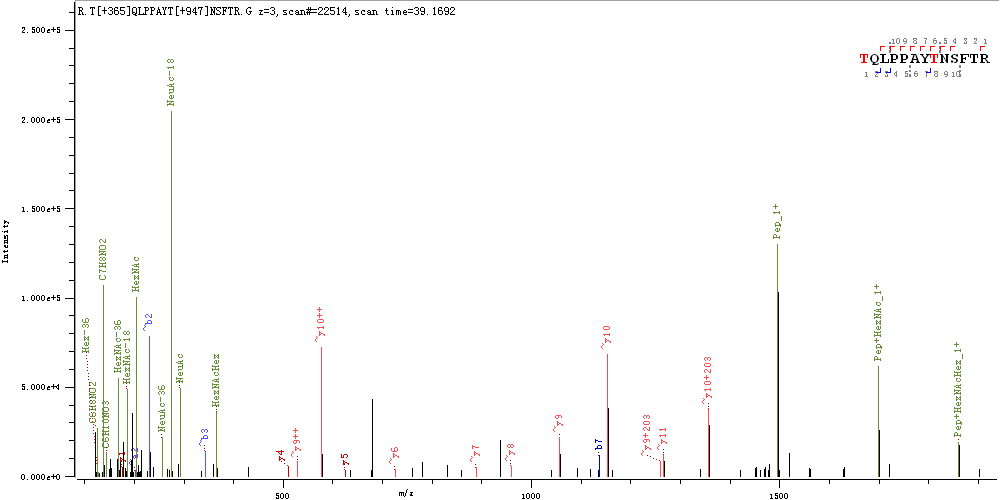


**S31**


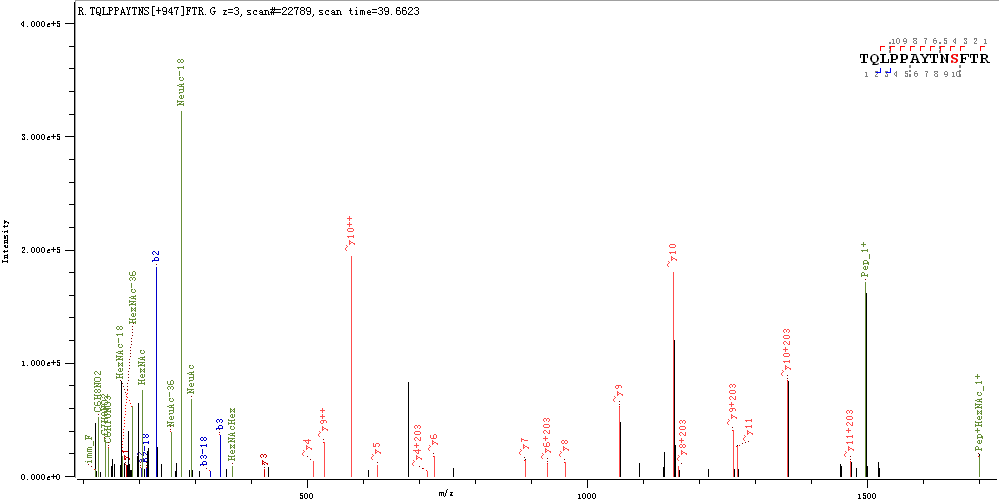


**T33**


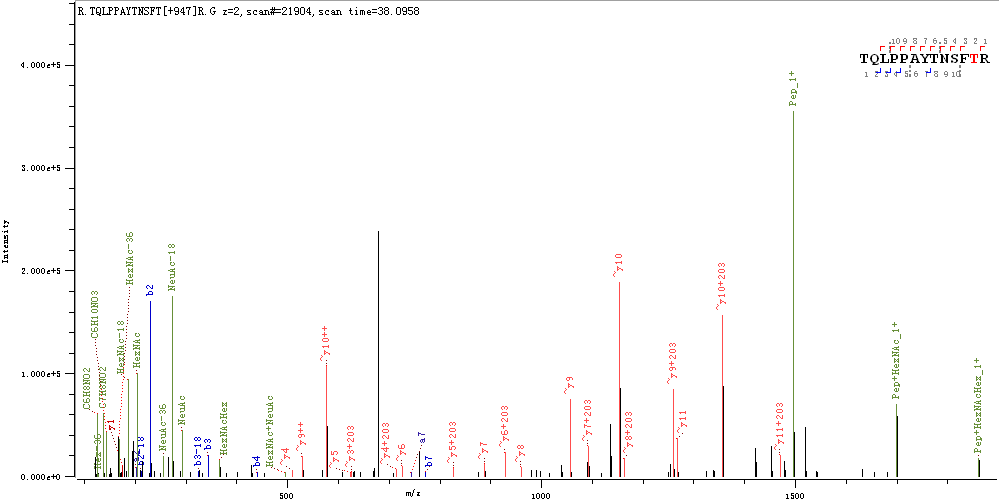


**T124**


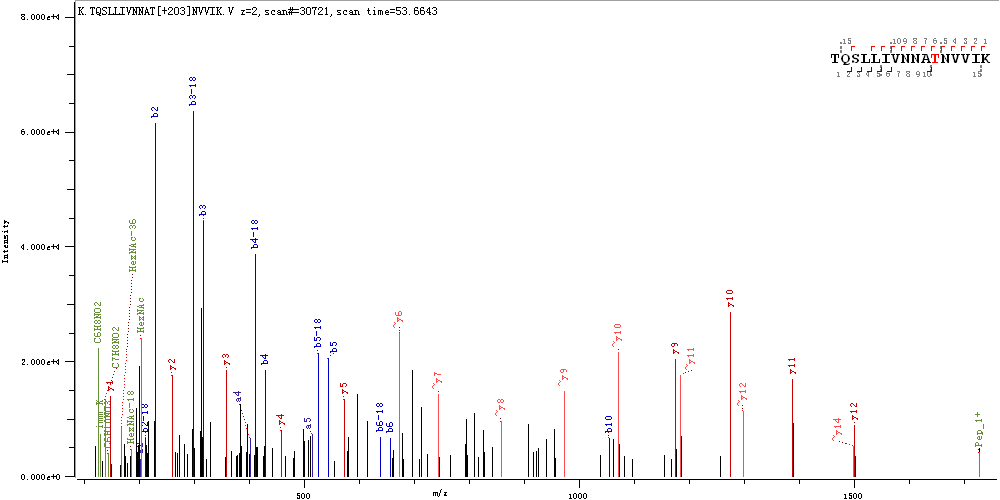


**T284 & T286**


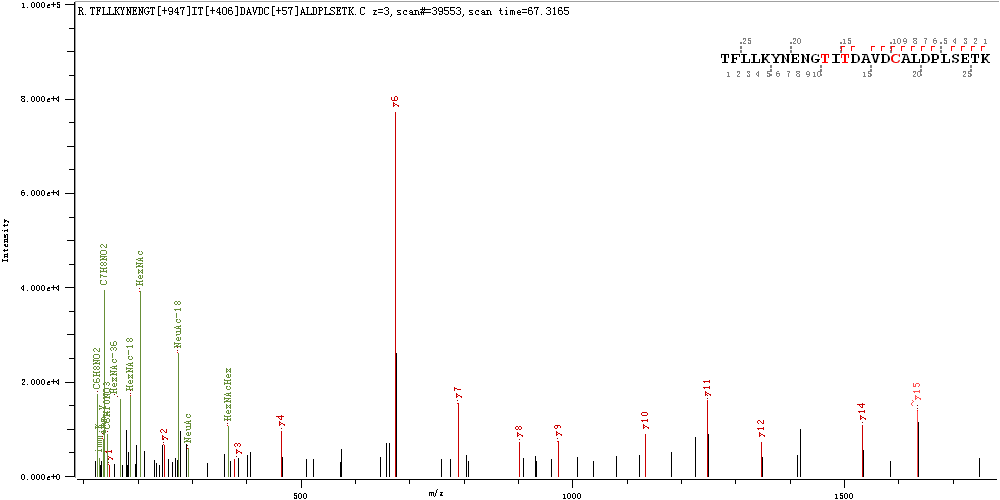


**S297 & T299**


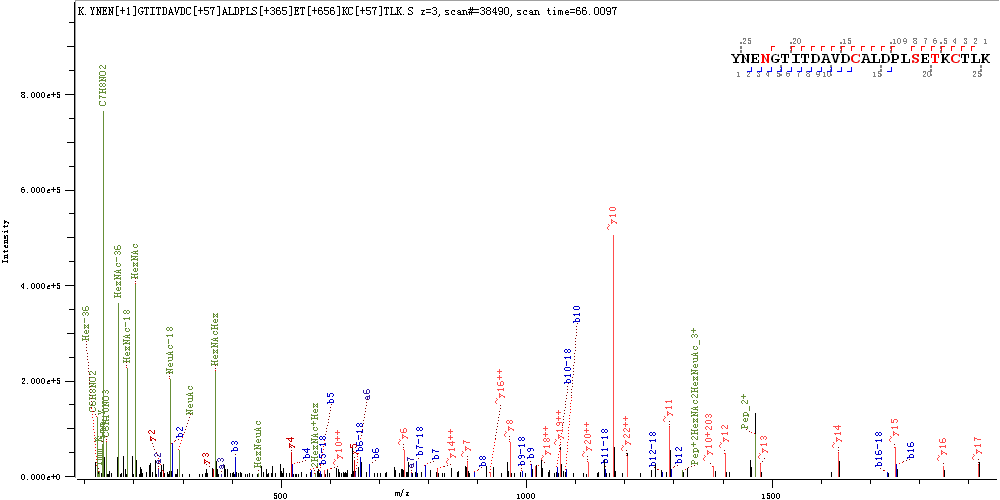


**T302**


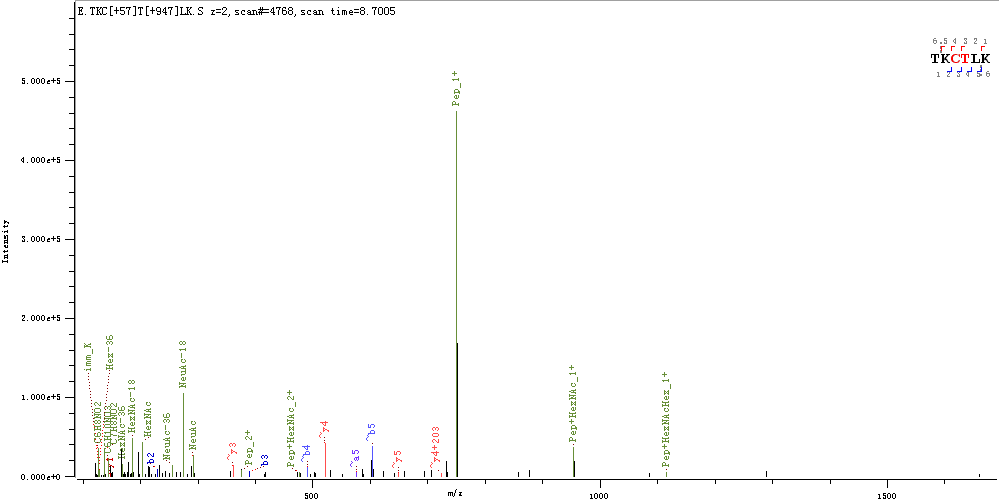


**S305 & T307**


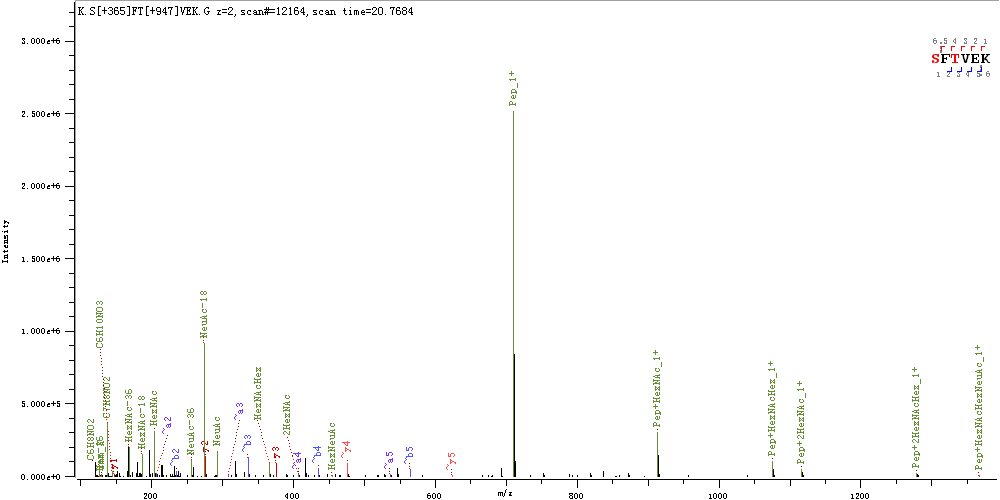


**T315**


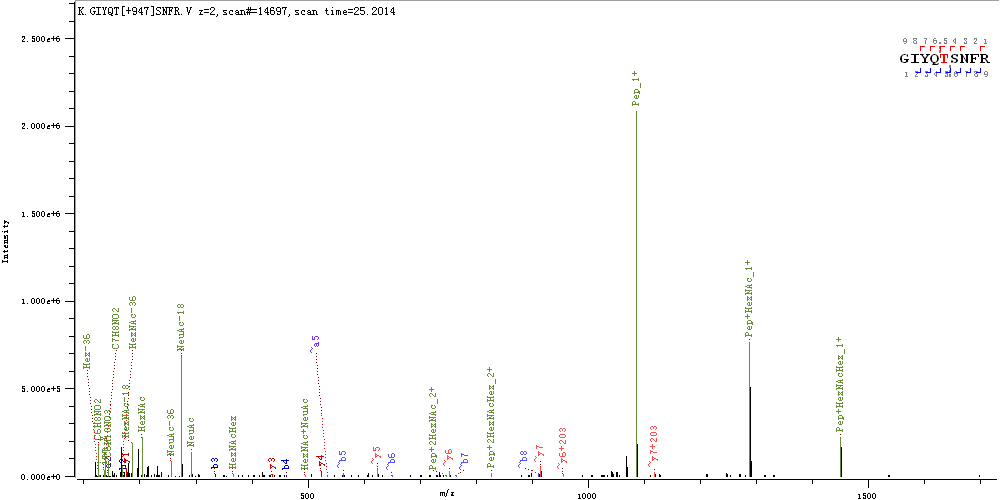


**T315 & S316**


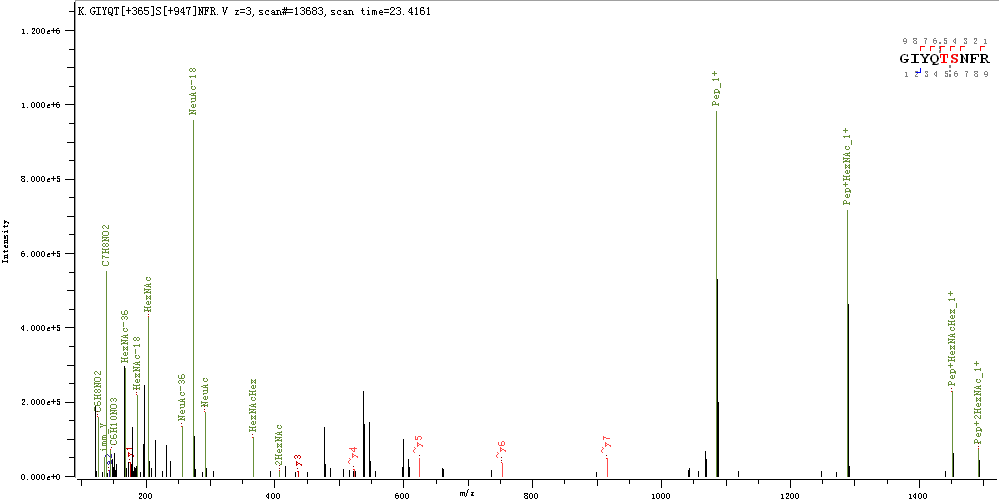


**T323 & S325**


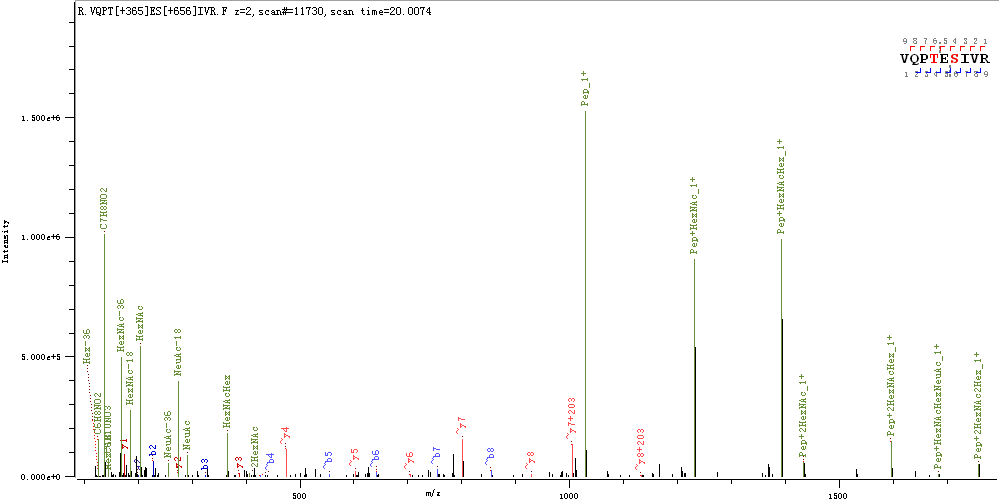


**T573 & T581**


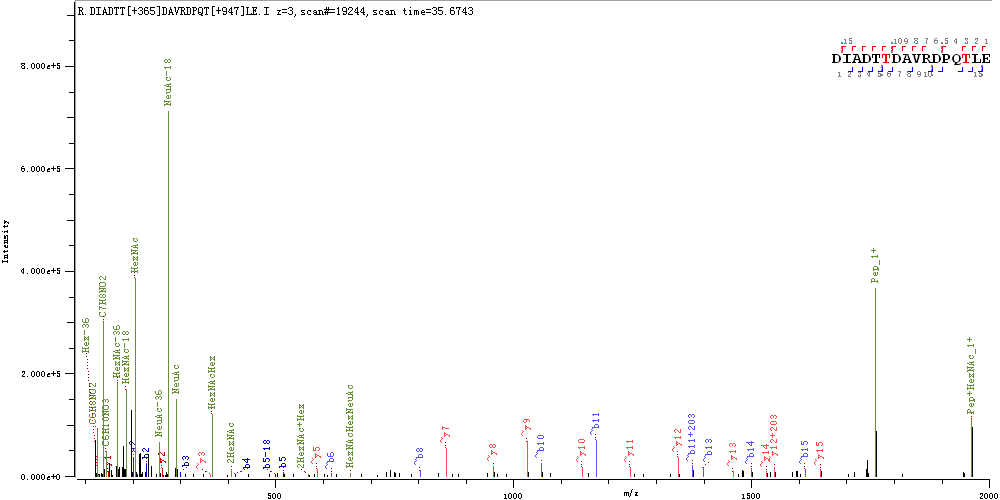


**T630 & T632**


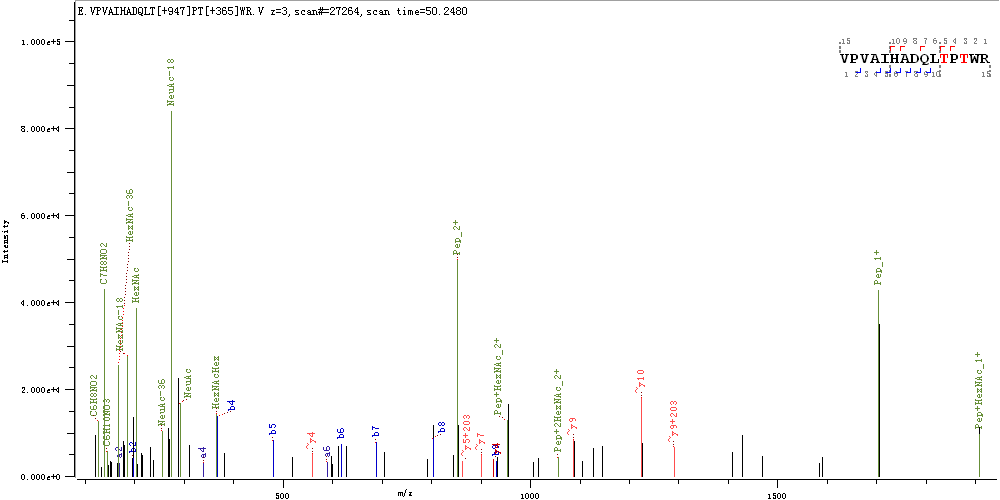


**S637 & T638**


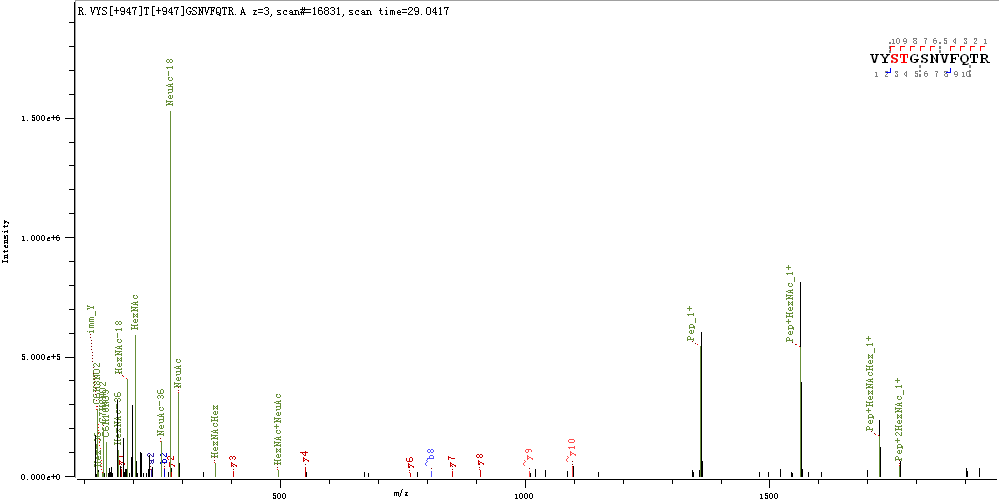


**S640**


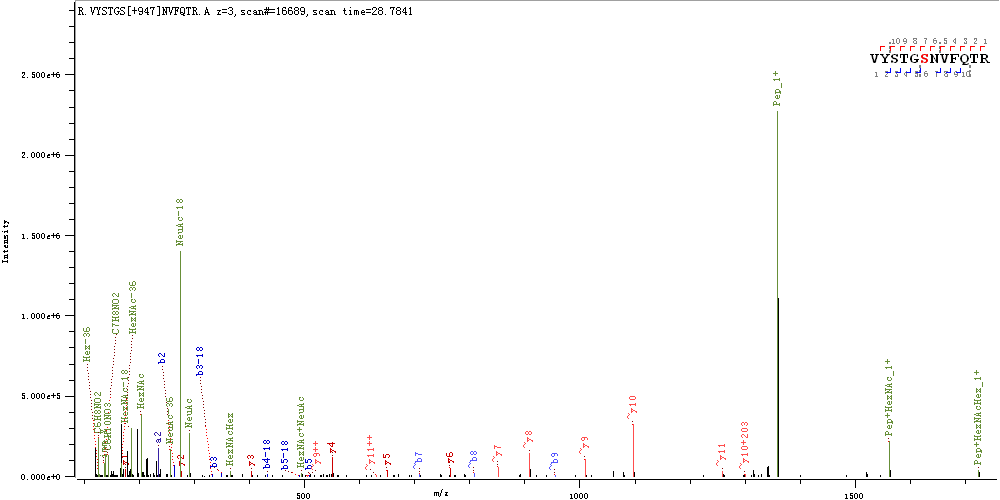


**T645**


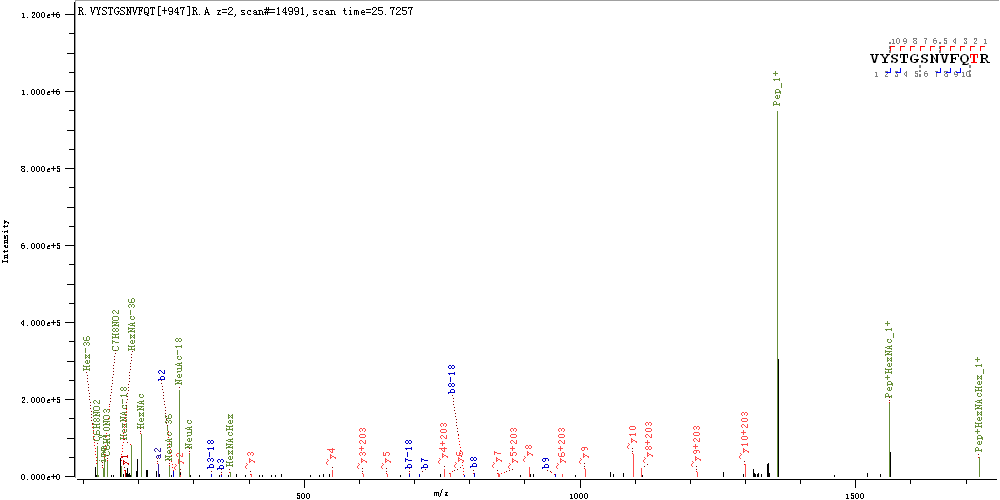


**T659**


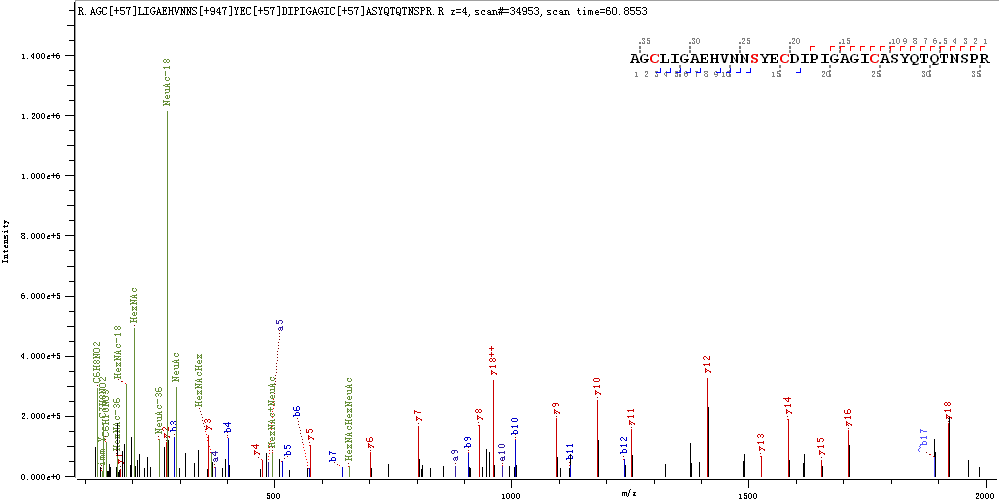


**S673 & S680**


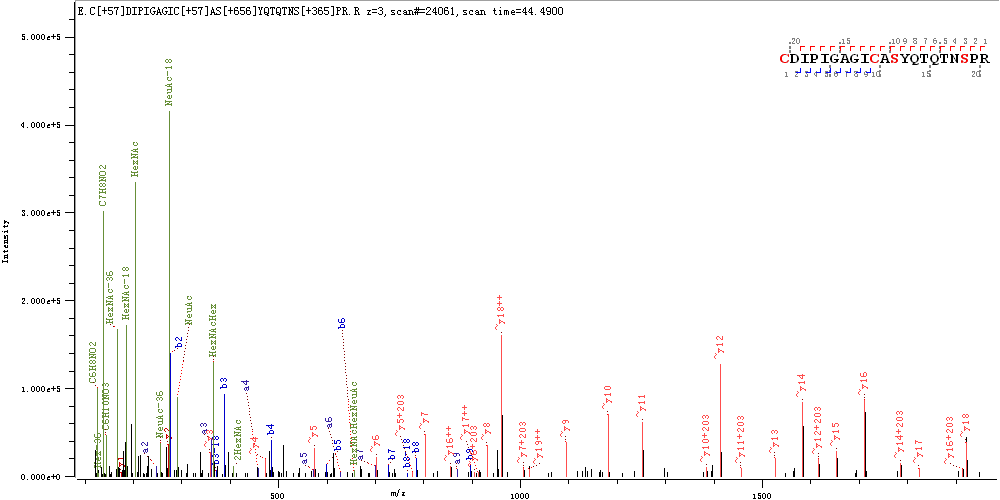


**T676 & S678**


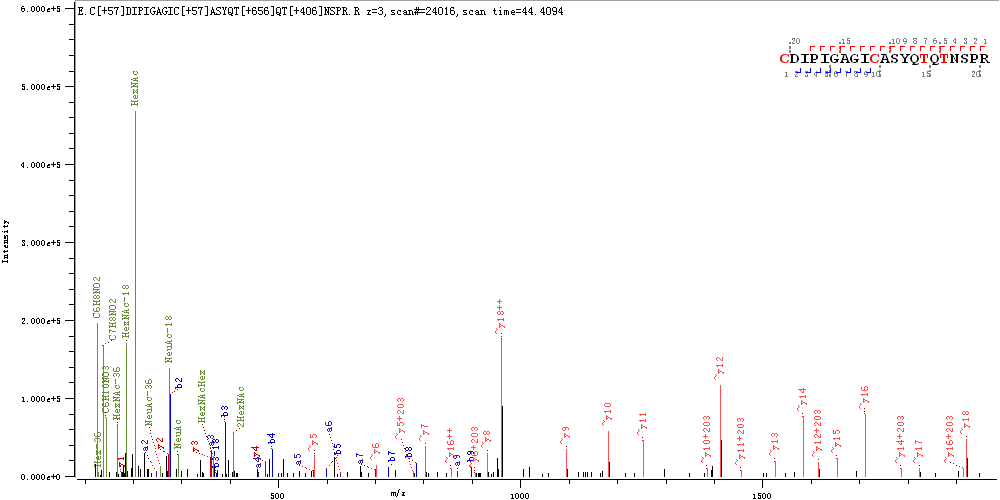


**Supplementary Figure S4.** Spectra of intact *O*-glycopeptides of SARS-CoV-2 S protein expressed in insect cells with ambiguously and unambiguously assigned *O*-glycosites by EThcD

**T95**


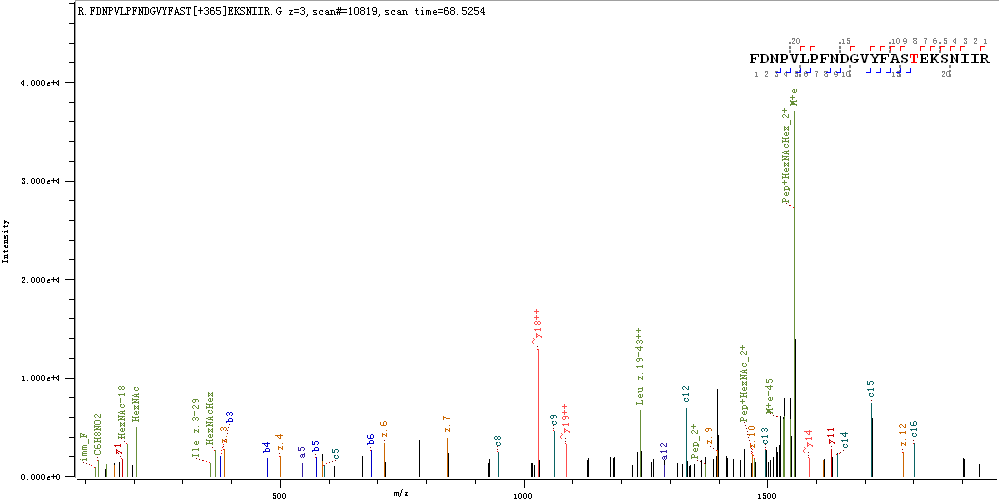


**T286**


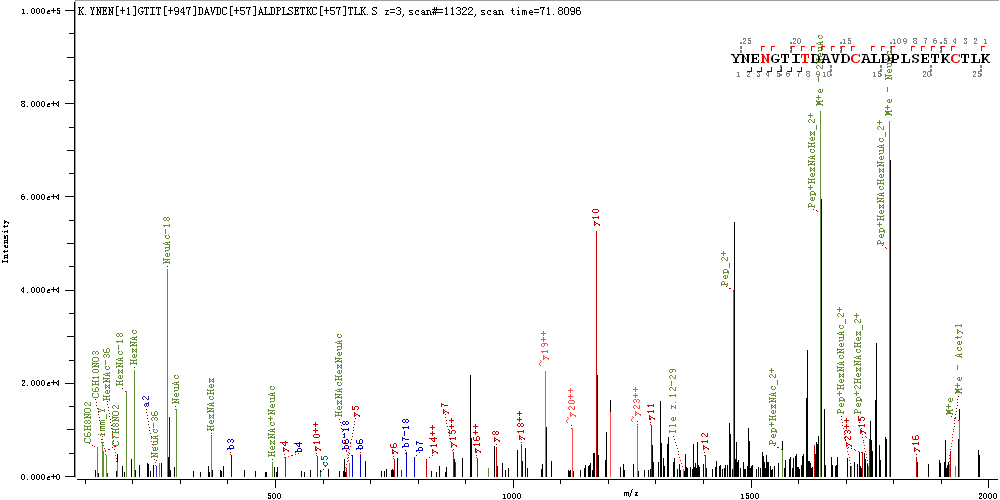


**S297&T299**


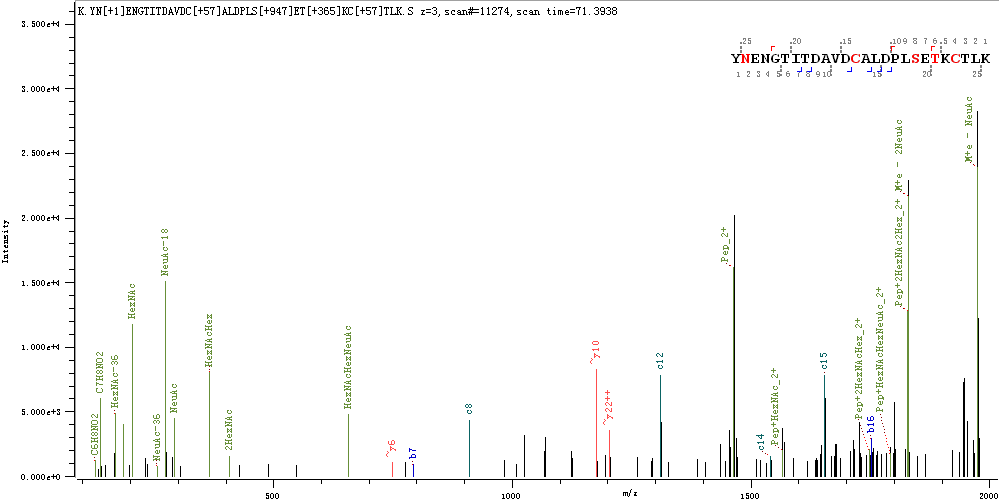


**T323**


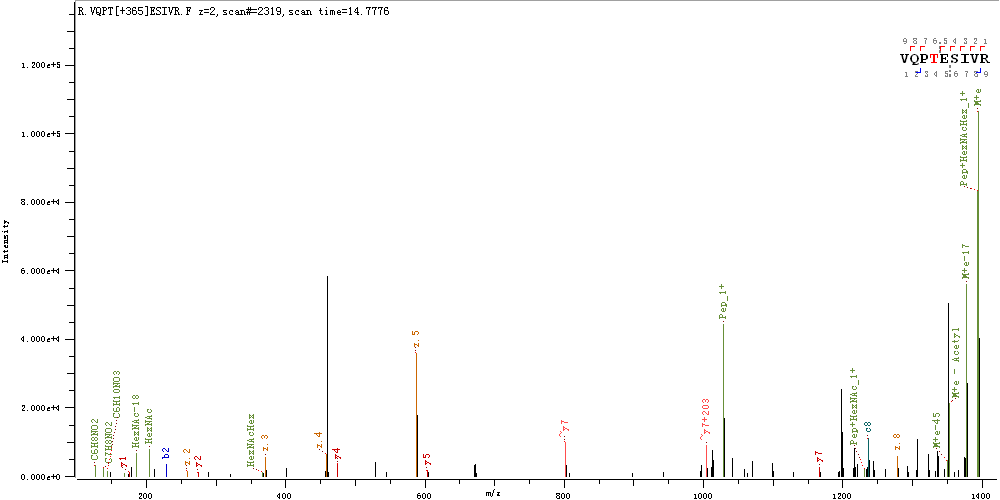


**T572**


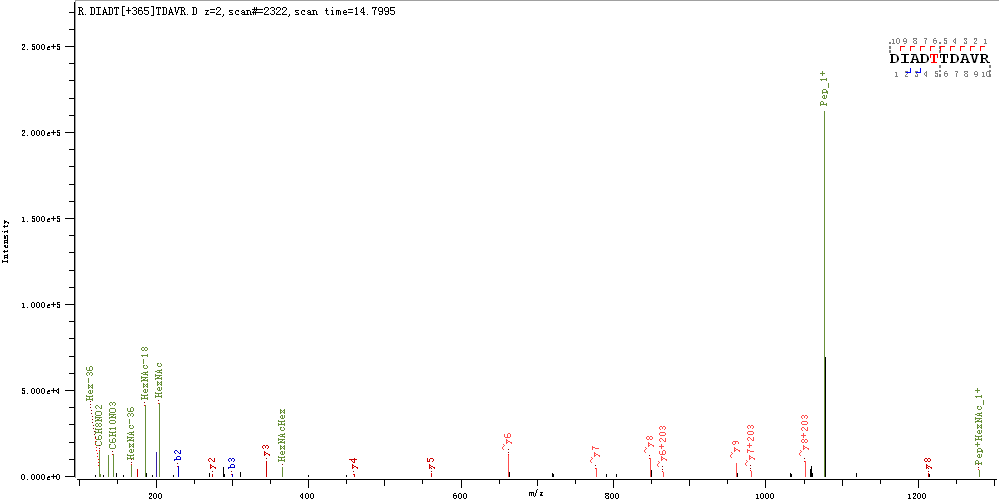


**T573**


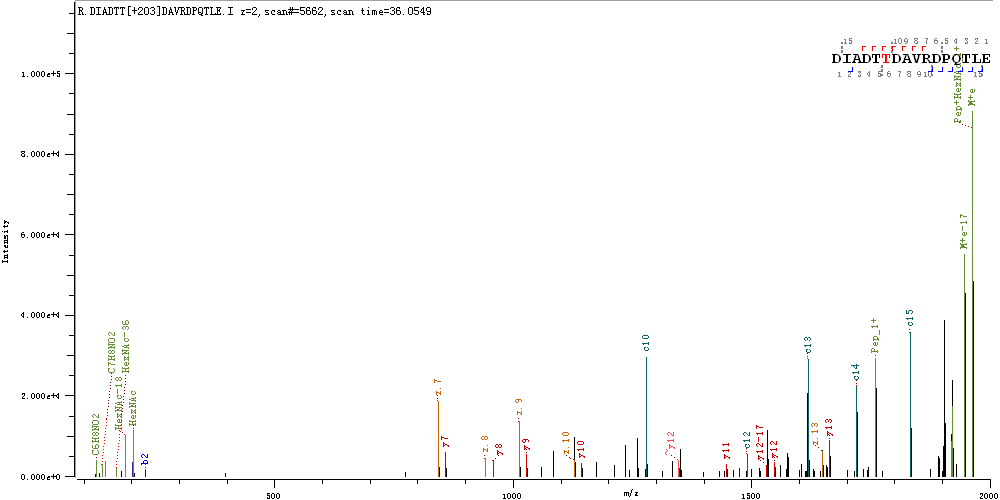


**S659& S673**

**
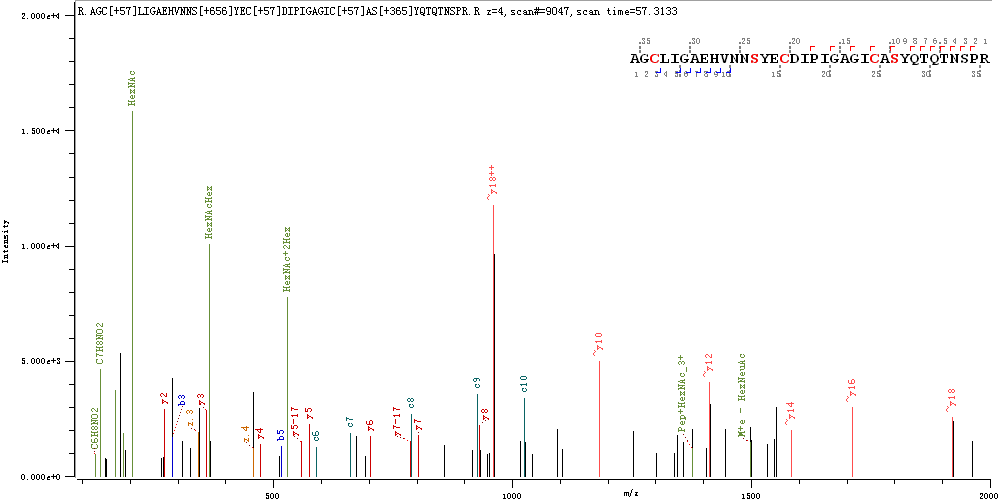
**

**S659 & T676**

**
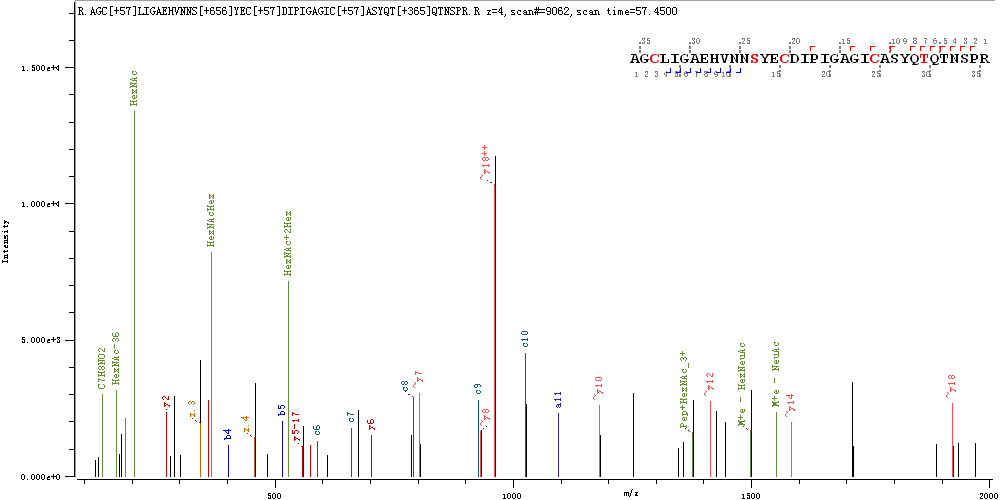
**

**T678 & S680**

**
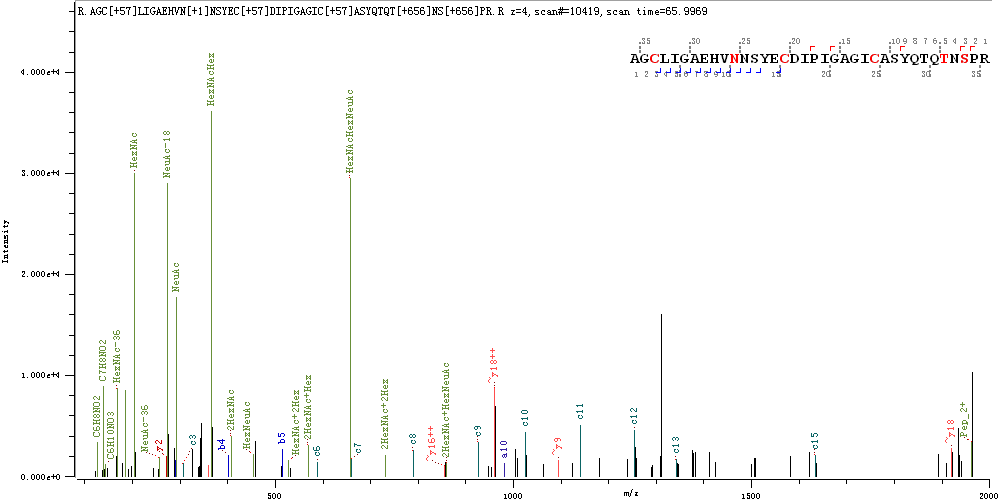
**

**Supplementary Figure S5.** Spectra of intact *O*-glycopeptides of SARS-CoV-2 S protein expressed in human cells with ambiguously and unambiguously assigned *O*-glycosites by EThcD

**T124**

**
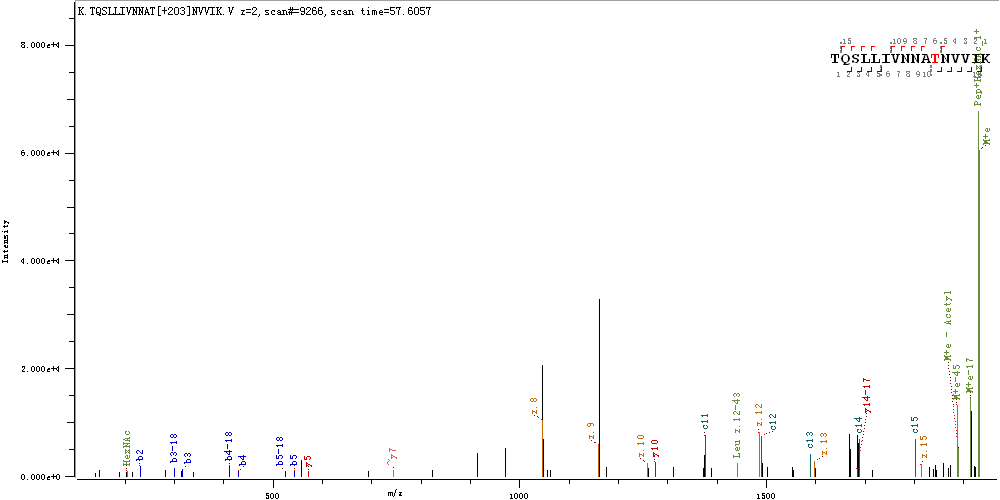
**

**T285**

**
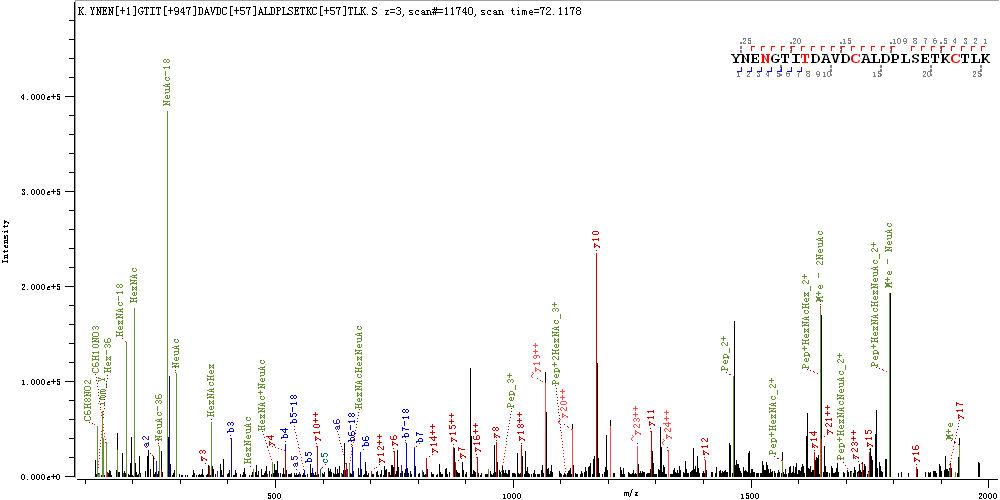
**

**S297 & T299**

**
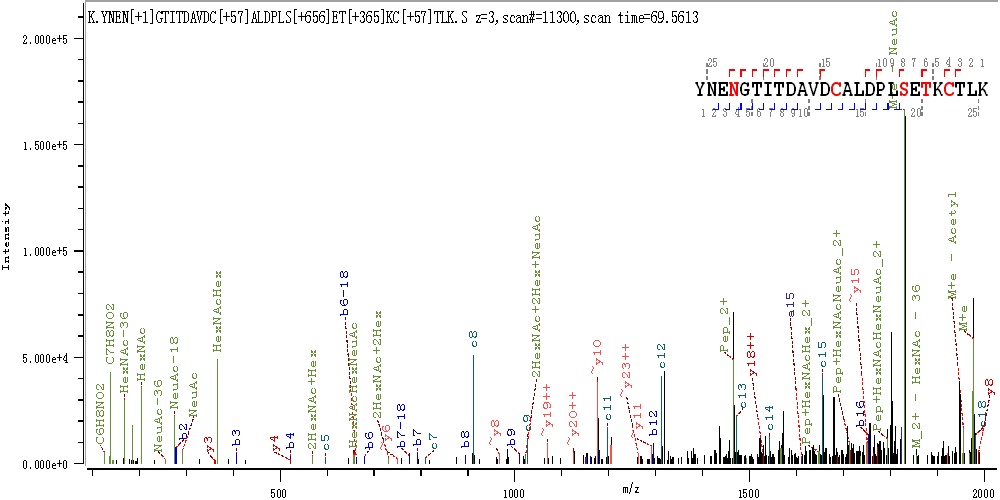
**

**T302**

**
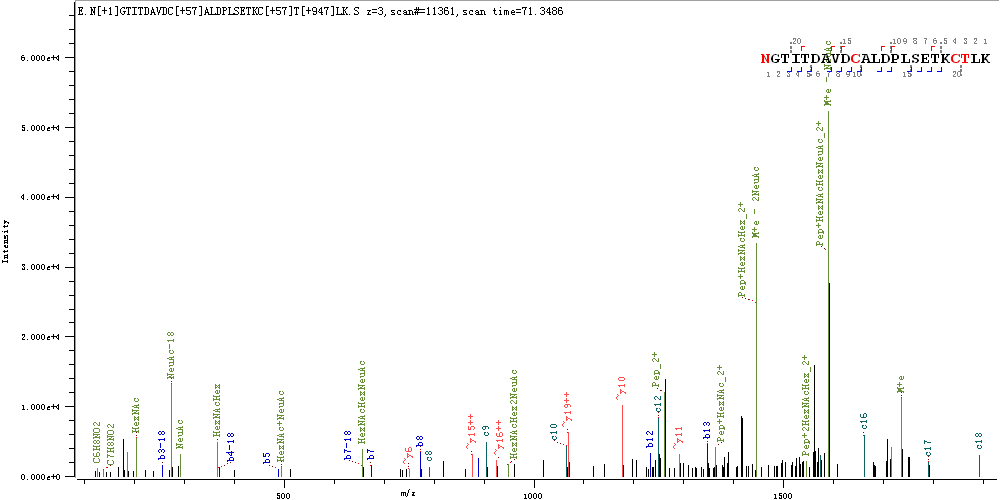
**

**T323**

**
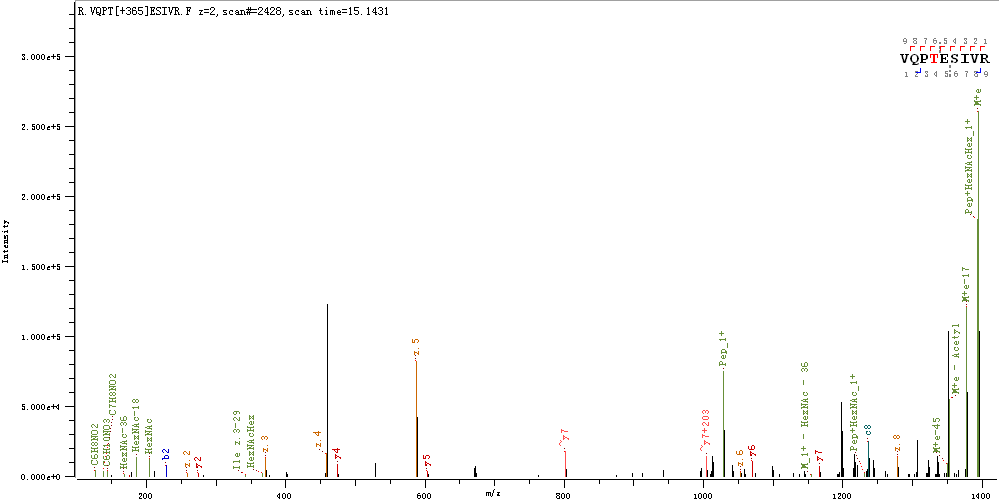
**

**T323 & S325**

**
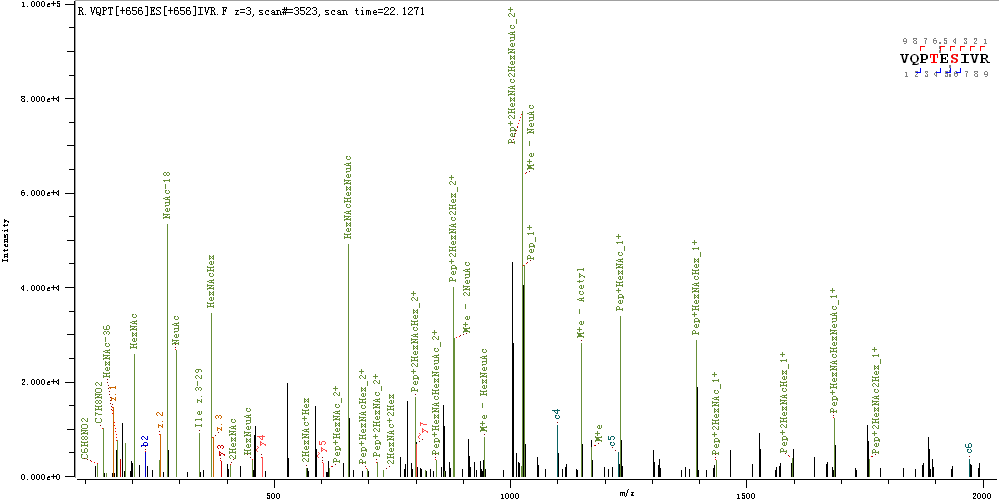
**

**T573**

**
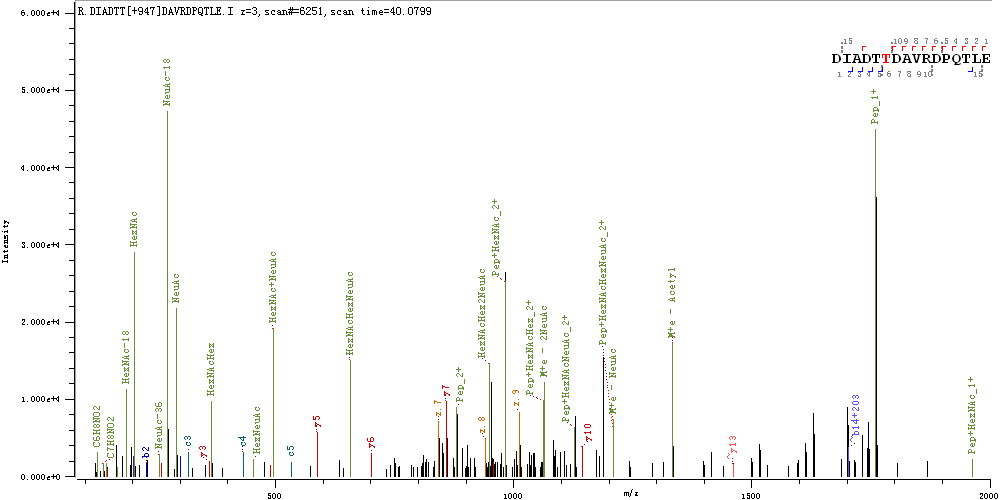
**

**T638**

**
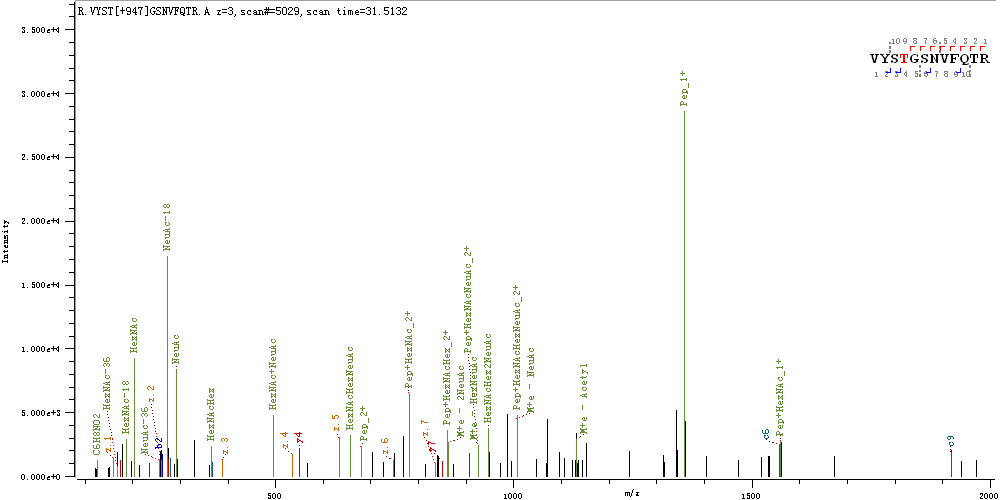
**

**S659 & T678**

**
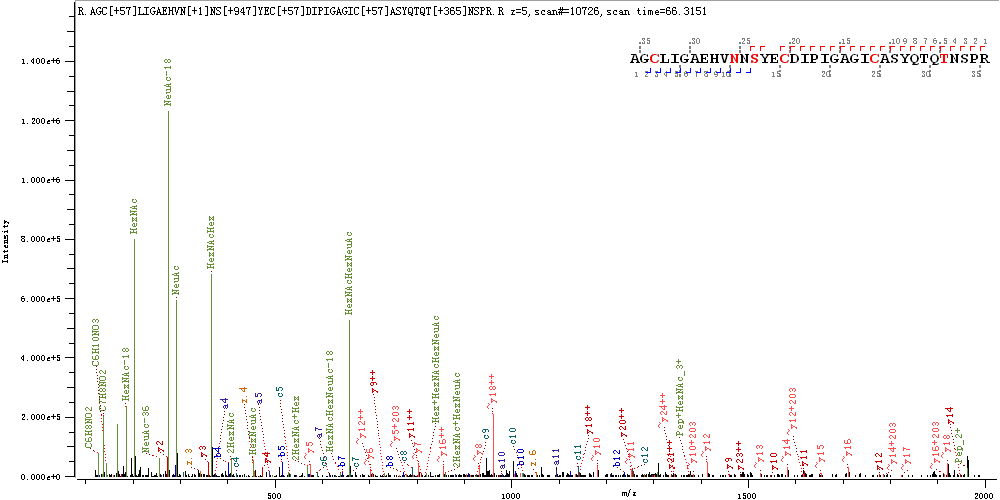
**

**S673 & T676**

**
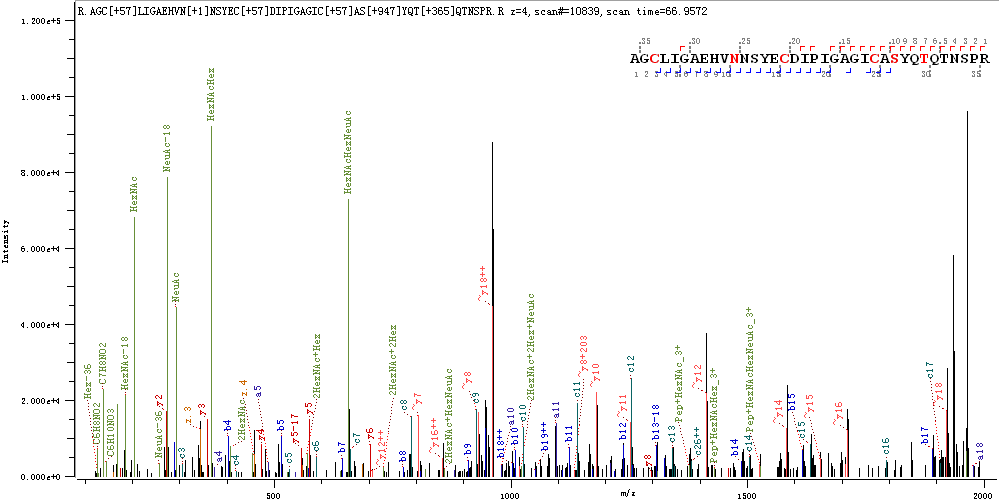
**

**T678 & S680**

**
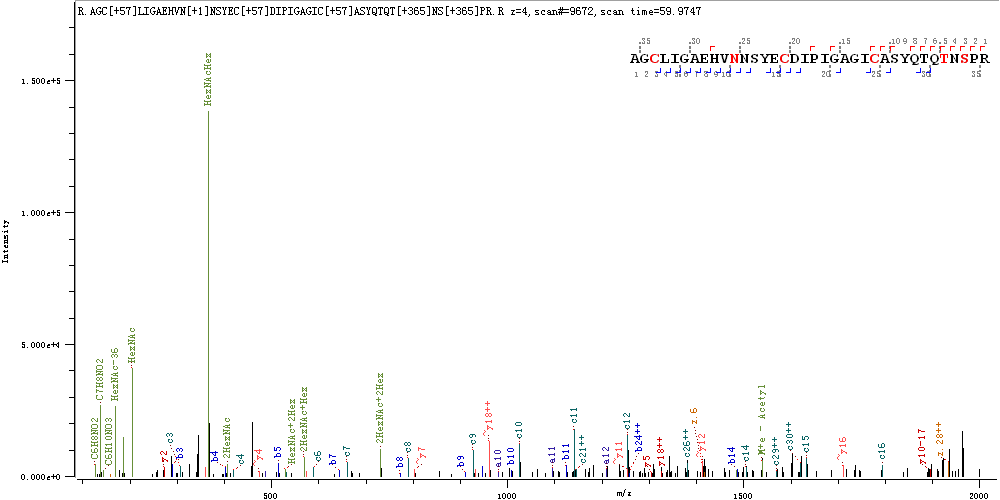
**
